# STA-9090 in combination with a statin exerts enhanced protective effects in rats fed a high-fat diet and exposed to diethylnitrosamine and thioacetamide

**DOI:** 10.3389/fphar.2024.1454829

**Published:** 2024-09-04

**Authors:** Amir Mohamed Abdelhamid, Sameh Saber, Rabab S. Hamad, Mustafa Ahmed Abdel-Reheim, Abousree T. Ellethy, Maha M. Amer, Mohamed R. Abdel-Hamed, Enas A. Mohamed, Syed Suhail Ahmed, Hossam A. Elsisi, Mostafa M. Khodeir, Abdullah S. Alkhamiss, AlSalloom A. A., Mawahib Ahmed Elawad Abu Elgasim, Zainab H. Almansour, Basem H. Elesawy, Elsayed A. Elmorsy

**Affiliations:** ^1^ Department of Pharmacology, Faculty of Pharmacy, Delta University for Science and Technology, Gamasa, Egypt; ^2^ Biological Sciences Department, College of Science, King Faisal University, Al Ahsa, Saudi Arabia; ^3^ Central Laboratory, Theodor Bilharz Research Institute, Giza, Egypt; ^4^ Department of Pharmaceutical Sciences, College of Pharmacy, Shaqra University, Shaqra, Saudi Arabia; ^5^ Department of Pharmacology and Toxicology, Faculty of Pharmacy, Beni-Suef University, Beni Suef, Egypt; ^6^ Department of Oral and Medical Basic Sciences, Biochemistry Division, College of Dentistry, Qassim University, Buraidah, Saudi Arabia; ^7^ Department of Anatomy, College of Medicine, Qassim University, Buraidah, Saudi Arabia; ^8^ Department of Anatomy and Embryology, Faculty of Medicine, Ain Shams University, Cairo, Egypt; ^9^ Department of Anatomy, Faculty of Medicine, Cairo University, Cairo, Egypt; ^10^ Department of Microbiology and Immunology, College of Medicine, Qassim University, Buraidah, Saudi Arabia; ^11^ Department of Pharmacology and Toxicology, College of Pharmacy, Qassim University, Buraidah, Saudi Arabia; ^12^ Department of Clinical Pharmacology, Faculty of Medicine, Zagazig University, Zagazig, Egypt; ^13^ Department of Pathology, College of Medicine, Qassim University, Buraidah, Saudi Arabia; ^14^ Department of Pathology, Faculty of Medicine, Cairo University, Cairo, Egypt; ^15^ Department of Family and Community Medicine, College of Medicine, Qassim University, Buraidah, Saudi Arabia; ^16^ Biological Sciences Department, College of Science, King Faisal University, Hofuf, Saudi Arabia; ^17^ Department of Pathology, College of Medicine, Taif University, Taif, Saudi Arabia; ^18^ Department of Pathology, Faculty of Medicine, Mansoura University, Mansoura, Egypt; ^19^ Department of Pharmacology and Therapeutics, College of Medicine, Qassim University, Buraidah, Saudi Arabia

**Keywords:** STA-9090, HSP90, HMG-CoA reductase, hedgehog pathway, inflammation/fibrosis, dyslipideamia

## Abstract

**Introduction:**

Liver fibrosis is a significant global health burden that lacks effective therapies. It can progress to cirrhosis and hepatocellular carcinoma (HCC). Aberrant hedgehog pathway activation is a key driver of fibrogenesis and cancer, making hedgehog inhibitors potential antifibrotic and anticancer agents.

**Methods:**

We evaluated simvastatin and STA-9090, alone and combined, in rats fed a high-fat diet (HFD) and exposed to diethylnitrosamine and thioacetamide (DENA/TAA). Simvastatin inhibits HMG-CoA reductase, depleting cellular cholesterol required for Sonic hedgehog (Shh) modification and signaling. STA-9090 directly inhibits HSP90 chaperone interactions essential for Shh function. We hypothesized combining these drugs may provide liver protective effects through complementary targeting of the hedgehog pathway. Endpoints assessed included liver function tests, oxidative stress markers, histopathology, extracellular matrix proteins, inflammatory cytokines, and hedgehog signaling components.

**Results:**

HFD and DENA/TAA caused aberrant hedgehog activation, contributing to fibrotic alterations with elevated liver enzymes, oxidative stress, dyslipidemia, inflammation, and collagen deposition. Monotherapies with simvastatin or STA-9090 improved these parameters, while the combination treatment provided further enhancements, including improved survival, near-normal liver histology, and compelling hedgehog pathway suppression.

**Discussion:**

Our findings demonstrate the enhanced protective potential of combined HMG CoA reductase and HSP90 inhibition in rats fed a HFD and exposed to DENA and TAA. This preclinical study could help translate hedgehog-targeted therapies to clinical evaluation for treating this major unmet need.

## 1 Introduction

Liver fibrosis stands out as a critical finding with substantial implications for both morbidity and mortality among patients. Various liver conditions, including fatty liver diseases, sustain continuous hepatocellular damage, ultimately resulting in the development of fibrosis (Berumen et al., 2021). Significantly, fibrosis emerges as the primary histopathological feature with the most profound impact on mortality (Stål, 2015). This progression inevitably leads to cirrhosis, accompanied by complications such as hepatocellular carcinoma (HCC) and liver failure (Abd El-Fattah et al., 2022). As a complication of cirrhosis, HCC claims the lives of approximately one million people globally each year, contributing to its status as the 16th most common cause of death (Nasr et al., 2023; Saber et al., 2023a; Saber et al., 2023b). Given this, the early diagnosis and suppression of liver fibrosis becomes paramount (Asrani et al., 2019). There are limited treatment options for advanced fibrosis. Liver transplantation stands as the exclusive viable therapeutic option for addressing the complications arising from liver fibrosis and cirrhosis. So, there is an urgent need for novel anti-fibrotic drugs to halt progression and complications.

A key driver of fibrogenesis is the activation of hepatic stellate cells (HSCs) into collagen-producing myofibroblasts, mediated by oxidative stress, inflammation, and profibrogenic cytokines (Friedman, 2003; Tsuchida and Friedman, 2017; Mohammed et al., 2023b; Elmorsy et al., 2024).

The Hedgehog (Hh) signaling pathway plays a crucial role in embryonic development and tissue homeostasis, and its dysregulation has been implicated in various pathological conditions, including liver fibrosis and HCC (Omenetti et al., 2011). The canonical Hh pathway is initiated when Hh ligands bind to the transmembrane receptor Patched (PTCH), relieving its inhibition on Smoothened (SMO). Activated SMO then triggers a signaling cascade that culminates in the activation of Gli transcription factors, which regulate the expression of Hh target genes (Briscoe and Thérond, 2013). In the context of liver disease, aberrant Hh signaling contributes to the activation of hepatic stellate cells, promoting fibrogenesis and disease progression (Syn et al., 2009). When activated in stromal cells of injured tissues, the Hedgehog pathway plays a crucial role in normal wound healing responses, influencing inflammation, vascular remodeling, and fibrogenesis. In the liver, sustained Hh signaling in stromal cells significantly contributes to the pathogenesis of cirrhosis (Dutta et al., 2023).

Our study focuses on two compounds that modulate the Hh pathway through distinct mechanisms. STA-9090 is a potent inhibitor of heat shock protein 90 (HSP90), a molecular chaperone essential for the stability and function of numerous oncoproteins, including components of the Hh pathway (Taipale et al., 2010). By disrupting HSP90 function, STA-9090 indirectly suppresses Hh signaling, potentially attenuating fibrogenic responses in the liver. Conversely, simvastatin (SMVT), an HMG-CoA reductase inhibitor primarily used for cholesterol reduction, has been shown to modulate Hh signaling by depleting cellular cholesterol, which is necessary for SMO activation (Lauth et al., 2010; Talreja et al., 2024). By targeting the Hh pathway through these complementary mechanisms, we hypothesize that combining STA-9090 and SMVT may offer enhanced therapeutic efficacy in mitigating liver fibrosis and associated pathologies.

In our current investigation, we employed diethylnitrosamine (DENA) and thioacetamide (TAA) within our rat model. Both substances are potent hepatotoxins that undergo metabolic activation, leading to the formation of toxic reactive metabolites. This process subsequently induces hepatocellular necrosis and the recruitment of inflammatory cells. The resulting necro-inflammatory response initiates the production of reactive oxygen species (ROS) and the release of proinflammatory cytokines, including tumor necrosis factor alpha (TNF-α) (Mehendale and Chilakapati, 2010; Hajovsky et al., 2012). These processes ultimately activate HSCs. These cells are the primary producers of collagen, contributing to progressive liver fibrosis that closely resembles the pathology observed in human diseases (Yanguas et al., 2016). Fat changes are observed in the liver tissue in the presence of a high-fat diet (HFD), which serves as an additional insult to the liver. We selected this model with the intention of closely replicating the actual hepatic environment of fatty changes, fibrosis, and early dysplastic alterations.

## 2 Methods

### 2.1 *In Vitro* analyses for evaluation of the synergistic activity in TGF-β-treated HepG2 cells

The HepG2 cell line from the American Type Culture Collection was cultured in Dulbecco’s modified Eagle’s medium with 10% fetal bovine serum (FBS), 100 μg/mL streptomycin, 100 IU/mL penicillin, and 2 mM glutamine. Regular passaging occurred and exponential growth phase cells were seeded into 96-well plates at 2 × 10^4^ cells/well in 100 μL BSA-free medium. Controlled conditions of temperature (37°C) and gas composition (21% O2, 74% N2, and 5% CO2) were maintained. After 24 h, during which the cells were starved, the cells were treated with transforming growth factor beta (TGF-β) (Abcam) (5 ng/mL) (Stopa et al., 2000; Liu et al., 2015) for 6 h. Then the medium was replaced with fresh 2% BSA containing-medium with 0, 2, 4, 8, or 16 µM SMVT or 0, 0.002, 0.004, 0.008, or 0.016 µM STA-9090 or combination (concentrations as per the dose-response matrix). Following 24-h incubation, an MTT assay assessed growth inhibition by measuring optical density at 490 nm using a Biotek plate reader (Vermont, United States of America) after formazan crystals complete dissolution in DMSO (Mourad et al., 2021). SynergyFinder 3.0 software was used to calculate combination synergy scores and to generate 2D/3D synergy maps based on interactions across concentrations (Ianevski et al., 2022). The extent of synergy or antagonism in drug combinations is measured by evaluating the observed response in terms of growth inhibition rate and comparing it to the anticipated response determined through the Zero Interaction Potency (ZIP) synergy model.

### 2.2 Rational for using TGF-β-treated HepG2 cells

Treating HepG2 cells with TGF-β can mimic the fibrogenic environment in the liver and induce epithelial-to-mesenchymal transition (EMT). A study found that TGF-β1 induced EMT in HepG2 cells, leading to morphological changes and a shift from an epithelial to a fibroblast-like morphology, along with changes in the expression of EMT markers (Liu et al., 2015). Additionally, as a transformed liver cell type, HepG2 mimics some characteristics of normal hepatocytes (Müsch, 2014) and allows evaluation of the direct toxicity of tested drugs towards liver cells. Hence, we employed TGF-β-treated HepG2 cells to evaluate the synergistic cytotoxic effect of STA-9090 and SMVT combined therapy.

### 2.3 Animal model and experimental groups

Male adult Sprague Dawley rats received 25 mg/kg intraperitoneal DENA, Sigma-Aldrich, St Louis, MO) at 14 days old, followed by 300 mg/L oral TAA in drinking water from 4–16 weeks old. Treatment groups received 30 mg/kg/day oral SMVT (MSD, Egypt) dispersed in 0.5% CMC solution and/or 10 mg/kg intraperitoneal STA-9090 (SelleckChem, Houston, TX, United States of America) dissolved in a DRD solution (10% DMSO, 18% Cremophor RH 40, 3.6% dextrose, and 68.4% water) every other day. The initiation of drug administration involving STA-9090 and/or SMVT took place in parallel with TAA therapy. Rats also received HFD in parallel with TAA therapy (at week 4). All control animal cohorts were subjected to identical dosing schedules and administered the vehicle.

**FIGURE 1:**
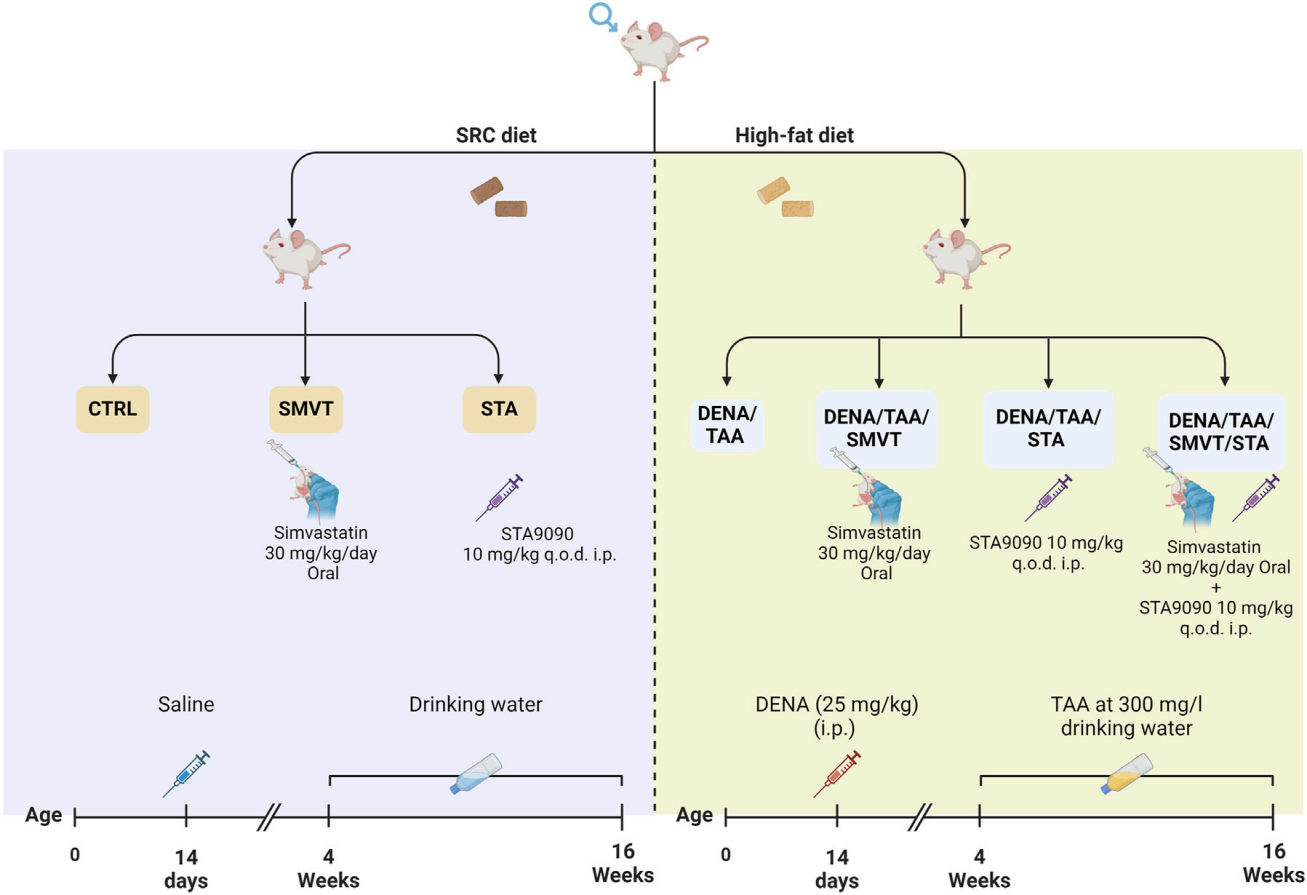
In the experimental protocol, rats were randomly assigned to seven groups: CTRL as the normal control, SMVT as the SMVT control, and STA as the STA-9090 control, all of which received a SRD. The DENA/TAA group was given DENA/TAA and HFD. The DENA/TAA/SMVT group received DENA/TAA, HFD, and SMVT, while the DENA/TAA/STA group received DENA/TAA, HFD, and STA-9090. The DENA/TAA/SMVT/STA group was treated with DENA/TAA, HFD, and a combination of SMVT and STA. After the study, rats were euthanized by decapitation following anesthesia, and blood was collected, and livers were excised.

The rats were randomly assigned to seven groups as illustrated in Figure 1: the CTRL group (n = 6) served as the normal control; the SMVT group (n = 6) acted as the control group for SMVT; the STA group (n = 6) functioned as the control group for STA-9090. The normal control and both drug control groups received a standard rodent chow diet. The DENA/TAA group (n = 14) was exposed to DENA/TAA and received a HFD. The DENA/TAA/SMVT group (n = 14) received DENA/TAA and the HFD along with SMVT. The DENA/TAA/STA group (n = 14) received DENA/TAA, the HFD, and STA-9090. The DENA/TAA/SMVT/STA group (n = 14) received DENA/TAA and the HFD along with a combined therapy of SMVT and STA. Upon completion of the study, rats were euthanized by decapitation after anesthesia induction using a mixture of 12.5 mg/kg Xylazine and 87.5 mg/kg Ketamine. After euthanasia, blood was collected by cardiac puncture, and livers were excised. All procedures were ethically approved by the ethical committee of DU, Egypt, and conducted in strict compliance with relevant laws and regulations (Approval No: 16/2022,6).

#### 2.3.1 Diet composition

The standard rodent chow diet was formulated with 20% of its energy originating from protein, 67% from carbohydrates, and 13% from fat. In contrast, the HFD was designed with 20% of its energy sourced from protein, 35% from carbohydrates, and 45% from fat (Lomba et al., 2010; Yeung, 2015; Keshk et al., 2020).

#### 2.3.2 Rational of drug dosing

The animal model used in our study was previously reported by Henderson et al. (2018) where DENA was intraperitoneally administered at a dosage of 25 mg/kg body weight at 14 days of age. Upon reaching 4 weeks of age, the animal group received TAA at a concentration of 300 mg/L in their drinking water, while another was provided with a HFD. In this study, the authors aimed to study the pathological changes induced by DENA/TAA/HFD and found that the severity of hepatic damage was greater after multiple liver insults. Pathophysiologically, the animal model described induces significant liver damage, leading to chronic liver injury characterized by steatosis, fibrosis, and inflammation. The model culminated in a pro-tumorigenic environment in the context of chronic liver inflammatory disease with the intention of closely replicating the actual hepatic environment of fatty changes, fibrosis, and early dysplastic alterations.

STA-9090 was solubilized in a DRD solution, following established protocols (Scutigliani et al., 2022), and administered based on the dosing regimen outlined in a prior study (Mohammed et al., 2023a). SMVT, in previous research, was administered to rats at varying doses between 10 and 50 mg/kg/day (Sironi et al., 2003; Baytan et al., 2008; Kalonia et al., 2011; Mohamadin et al., 2011; Yildiz et al., 2014; Shalaby et al., 2017).

### 2.4 Blood and tissue sampling

After inducing anesthesia, blood samples were obtained *via* cardiac puncture, and the serum was promptly isolated for immediate biochemical analysis. Concurrently, the livers were meticulously harvested, rinsed to remove blood traces, and weighed. Subsequently, the livers underwent various processing methods: some samples were sectioned and preserved in 10% formalin for subsequent histopathological examination, while others were stored in RNA later for qRT-PCR analysis. Additionally, certain liver samples underwent immediate biochemical assessments, while others were snap-frozen and stored at −80°C for subsequent analyses.

### 2.5 Histological examination and immunohistochemistry evaluation

Histological examination and histopathological scoring of liver tissues were conducted systematically, incorporating both hematoxylin and eosin (H&E) staining for morphological assessment and Sirius Red staining to visualize collagen fibers, indicative of fibrosis. Following fixation in 10% formalin, the tissue samples were embedded in paraffin and sectioned into 5-μm slices (Youssef et al., 2022). A histopathologist blindly examined samples using a light microscope (Olympus CX23, Tokyo, Japan). The fibrosis area was computed using ImageJ software. The histopathological findings and the histopathological score followed the criteria established by Ishak et al. (1995). To assess hepatic stellate cell activation and the presence of myofibroblasts, immunohistochemistry targeting alpha-smooth muscle actin (α-SMA) was conducted. Antibodies for α-SMA were sourced from Thermo Fisher Scientific (Rockford, IL, United States of America). The expression of α-SMA was quantified by determining the percentage of the positive area in relation to the total area. This quantification was performed using ImageJ 1.53f software, precisely analyzing the extent of α-SMA expression in the examined tissue samples.

### 2.6 Assessment of liver function enzymes, albumin, total cholesterol (TC), and triglycerides (TG)

Serum levels of alanine aminotransferase (ALT), aspartate aminotransferase (AST), and alkaline phosphatase (ALP) were evaluated using kits obtained from Biodiagnostic (Giza, Egypt) while gamma-glutamyl transferase (γ-GT) was evaluated using a kit obtained from Sigma-Aldrich (St Louis, MO). Additionally, the determination of albumin, total cholesterol (TC), and triglycerides (TG) in serum samples was carried out using commercially available kits from Spectrum Diagnostics (Cairo, Egypt). The processing of serum samples followed the manufacturer’s instructions for each specific assay.

### 2.7 Evaluation of oxidative stress and antioxidant status

Fresh liver tissue samples were collected, weighed, and homogenized on ice in the isotonic buffer (50 mM potassium phosphate buffer, pH 7.5). Following centrifugation (1,600 × g for 15 min), the resulting supernatant was utilized to evaluate oxidative stress by measuring malondialdehyde (MDA) levels using the method by Mihara and Uchiyama (1978). Briefly, the supernatant was mixed with trichloroacetic acid (El-Nasr Chemical Co., Cairo, Egypt) and thiobarbituric acid (Sigma-Aldrich) and heated, before adding n-butanol and measuring absorbance at 535 nm. Results were expressed as µmol MDA per Gram tissue.

To assess antioxidant status, reduced glutathione (GSH) levels were determined based on the reduction of 5,5′-dithiobis (2-nitrobenzoic acid) and measuring absorbance at 405 nm using a kit provided by Biodiagnostic (Giza, Egypt). Glutathione peroxidase (GPx) activity was evaluated by measuring NADPH consumption in enzyme-coupled reactions at 340 nm. GPx units were calculated using a standard curve. Superoxide dismutase (SOD) activity was determined after erythrocyte elimination and tissue homogenization. Samples were incubated with reagents and absorbance was measured at 450 nm. All spectrophotometric assays for parameters of oxidative stress and antioxidant status were performed in duplicate using standard protocols and commercial reagents.

Myeloperoxidase (MPO) as an indicator of inflammation and neutrophil infiltration was assayed in liver tissue homogenate in ice-cold PBS (0.01M, pH = 7.4) using a glass homogenizer on ice followed by centrifugation for 5 min at 5,000 × g to retrieve the supernatant. Samples assayed as per the instructions given by Biovision (CA, United States of America).

### 2.8 Assessment of hepatic hydroxyproline

The hydroxyproline content in liver tissues was quantified as an indicator of collagen deposition using the previously described method (Reddy and Enwemeka, 1996). Briefly, liver homogenate aliquots underwent acid hydrolysis at 120°C for 30 min in sodium hydroxide. After adding chloramine T solution and incubating at room temperature to allow oxidation, Ehrlich’s reagent was added and heated to develop a colored complex. Following cooling, the absorbance was measured spectrophotometrically at 550 nm. The resulting hydroxyproline levels were expressed as micrograms per Gram of liver tissue. All assays were performed in duplicate using standard protocols. The hydroxyproline measurement quantitatively represents collagen accumulation as a marker of fibrosis progression in the experimental liver tissues.

### 2.9 Analysis of gene expression by quantitative PCR (qRT-PCR)

Total RNA was extracted from rat liver samples using the Qiagen RNeasy Mini Kit. cDNA was synthesized, and quantitative real-time PCR was performed using the QuantiTect SYBR Green PCR Kit per the manufacturer’s instructions. The comparative Ct method was used to determine relative mRNA levels of target genes, with β-actin as the internal control. The fold change in the expression of each target gene was calculated using the formula for relative quantification (RQ): RQ = 2^(−ΔΔCt)^. Primer sequences were: β-actin forward 5′-TCA​CCC​ACA​CTG​TGC​CCA​TCT​ATG​A-3′, reverse 5′-CAT​CGG​AAC​CGC​TCA​TTG​CCG​ATA​G-3′; collagen type I alpha 1 (Col1a1) forward 5′-GAC​ATG​TTC​AGC​TTT​GTG​GAC​CC-3′, reverse 5′-AGG​GAC​CCT​TAG​GCC​ATT​GTG​TA-3′; transforming growth factor β1 (TGF-β1) forward 5′-CTT​CTC​CAC​CAA​CTA​CTG​CTT​C-3′, reverse 5′-GGG​TCC​CAG​GCA​GAA​GTT-3′; GLI1 forward 5′-CAG​GGA​AGA​GAG​CAG​ACT​GA-3′, reverse 5′-CAG​GAG​GAT​TGT​GCT​CCA-3′; smoothened (SMO) forward 5′-GCC​TGG​TGC​TTA​TTG​TGG-3′, reverse 5′-GGT​GGT​TGC​TCT​TGA​TGG-3′; GLI2 forward 5′-ATA​AGC​GGA​GCA​AGG​TCA​AG-3′, reverse 5′-CAG​TGG​CAG​TTG​GTC​TCG​TA-3′. Triplicate reactions, including no-template controls, were performed.

### 2.10 Analysis of TNF-α, PDGF-BB, TIMP-1, TGF-β, shh, SMO, GLI1, GLI2, HSP90, and HSP70

Liver tissue samples were rinsed in cold PBS and homogenized. After centrifugation, the resulting supernatants were used to quantify levels of proteins by ELISA as per the manufacturer’s protocols for tumor necrosis factor-alpha (TNF-α; CUSABIO, Wuhan, China), platelet-derived growth factor-BB (PDGF-BB; Wuhan Fine Biotech Corp), tissue inhibitor of metalloproteinase-1 (TIMP-1; RayBiotech, GA, United States of America), transforming growth factor-beta (TGF-β; MyBioSource), Shh (Novus Biologicals, CA, United States of America), SMO (MyBioSource, CA, United States of America), GLI1 (Fine Test, Wuhan Fine Biotech Corp., Wuhan, China), GLI2 (Cloud-Clone Corp.), HSP90 (Novus Biologicals), and HSP70 (MyBioSource). Total protein was quantified by bicinchoninic acid (BCA; Thermo Fisher Scientific Inc., Rockford, United States of America) assay for normalization. All assays were performed in duplicates.

### 2.11 Statistical analysis

GraphPad Prism software (version 9.5.1, GraphPad Software, CA, United States of America) was utilized for statistical analyses to determine differences between experimental groups. Parametric data was analyzed by one-way ANOVA followed by Tukey’s *post hoc* tests, while non-parametric data was assessed by Kruskal–Wallis followed by Dunn’s *post hoc* tests. Results were expressed as mean ± standard deviation or median ± interquartile range (IQR) for parametric and non-parametric data respectively. Significance was considered at *p* ≤ 0.05. Kaplan-Meier survival plots graphically depicted cumulative survival over time, contrasting treatment groups to untreated control. Mantel-Cox log-rank tests compared survival between groups.

## 3 Results

### 3.1 STA/SMVT combination therapy resulted in a synergistic cytotoxic effect in TGF-β-treated HepG2 cells

**FIGURE 2:**
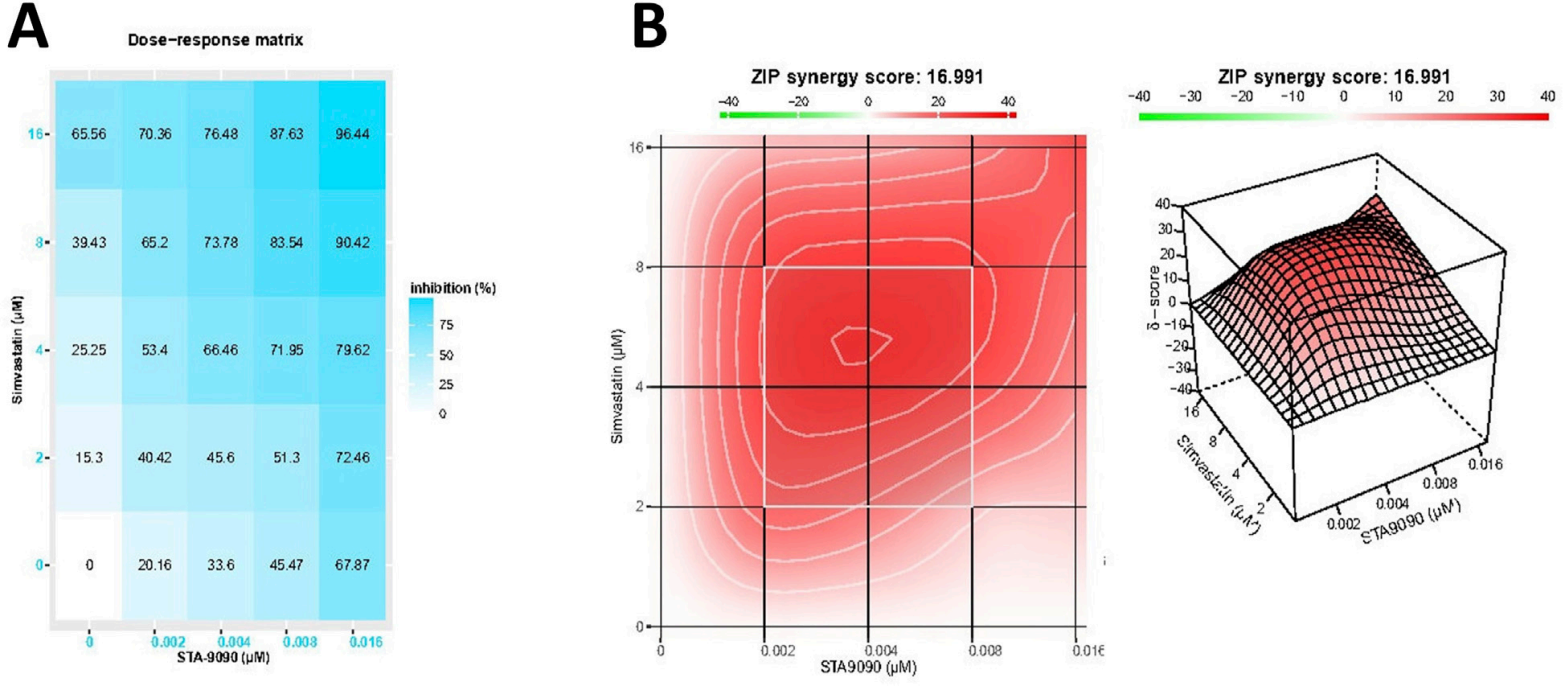
The matrix of growth inhibition rates (responses) and the 2D and 3D synergy maps. Matrix **(A)** illustrates the growth inhibition rates (responses) observed in TGF-β-treated HepG2 cells after 24 h of incubation with different concentrations of SMVT and STA-9090, both administered individually and in combination. The corresponding plot in Figure **(B)** represents the analysis using the ZIP synergy model, indicating a synergy score of 16.99 for the combined therapy. This synergy is visually depicted in the 2D and 3D synergy maps.

In Figure 2A, the dose-response matrix illustrates the cytotoxic response (growth inhibition rate) of TGF-β-treated HepG2 cells after 24-h incubation with varying concentrations of SMVT and STA-9090 combinations. The synergy between these drug combinations was quantified using the ZIP synergy model, resulting in a synergy score of 16.99. The 2D and 3D synergy maps in Figure 2B, with synergistic regions highlighted in red, visually emphasize the synergistic effect. The synergy score of 16.99, exceeding 10, suggests a likely synergistic interaction between SMVT and STA-9090. This value, indicating an average excess response due to drug interactions, corresponds to 16.99% of the response beyond what would be expected.

### 3.2 STA/SMVT combination therapy prevented body weight loss and restored liver weight index in rats fed a HFD and exposed to DENA and TAA

**FIGURE 3:**
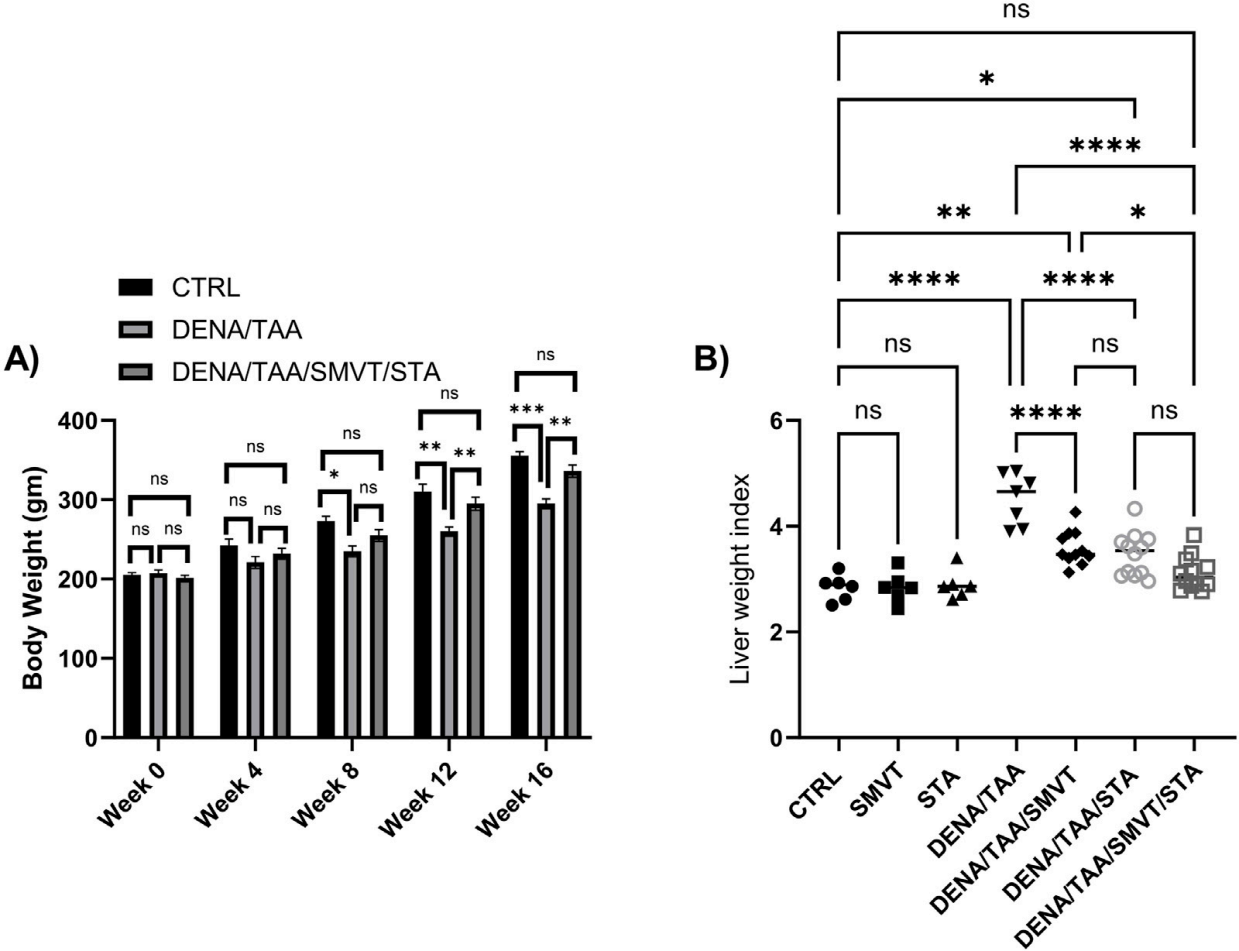
Effect of SMVT, STA-9090 (STA), and combination therapy on body weight **(A)**; and liver weight index **(B)** in rats fed a HFD and exposed to DENA and TAA. Data are presented as the mean ± SD. ns, non-significant, *p < 0.05, **p < 0.01, ***p < 0.001, ****p < 0.0001. CTRL, normal control group received the vehicle; SMVT, normal group received SMVT (30 mg/kg/day); STA, normal group received STA-9090 (10 mg/kg, i p. every other day); DENA/TAA, DENA/TAA-induced fibrosis group received the vehicle; DENA/TAA/SMVT, DENA/TAA-induced fibrosis group treated with SMVT (30 mg/kg/day); DENA/TAA/STA, DENA/TAA-induced fibrosis group treated with STA-9090 (10 mg/kg, i p. every other day); DENA/TAA/STA/SMVT, DENA/TAA-induced fibrosis group treated with SMVT (30 mg/kg/day) and STA-9090 (10 mg/kg, i p. every other day). The CTRL group and the drug control groups were provided with standard rodent chow whereas the diseased groups received AD.

The alteration in body weight was tracked every 4 weeks. The liver weight index, representing the ratio of liver weight to body weight, was calculated at the end of the experimental protocol. This index was computed by dividing the liver weight by the body weight, providing insight into the proportional changes in liver weight relative to the overall body weight over the course of the study. There were no significant differences in body weight between groups at baseline or after 4 weeks. However, starting at 8 weeks, the DENA/TAA group displayed significantly decreased body weight compared to CTRL, which continued through weeks 12 and 16. Treatment with SMVT/STA prevented the reduction in body weight at 12 and 16 weeks *versus* DENA/TAA (Figure 3A). The DENA/TAA group showed a significantly increased liver weight index compared to CTRL. All treatments significantly lowered the liver weight index compared to DENA/TAA. The combination therapy decreased the liver weight index to a level no longer significantly different from CTRL, representing the most effective treatment (Figure 3B).

### 3.3 STA/SMVT combination therapy enhanced cumulative survival in rats fed a HFD and exposed to DENA and TAA

**FIGURE 4:**
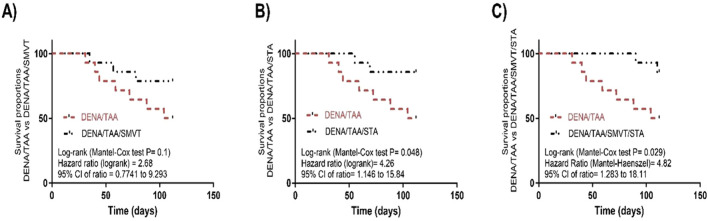
Effect of SMVT **(A)**, STA-9090 (STA) **(B)**, and combination therapy **(C)** on survival analysis in rats fed a HFD and exposed to DENA and TAA. DENA/TAA, DENA/TAA-induced fibrosis group received the vehicle; DENA/TAA/SMVT, DENA/TAA-induced fibrosis group treated with SMVT (30 mg/kg/day); DENA/TAA/STA, DENA/TAA-induced fibrosis group treated with STA-9090 (10 mg/kg, i.p. every other day); DENA/TAA/STA/SMVT, DENA/TAA-induced fibrosis group treated with SMVT (30 mg/kg/day) and STA-9090 (10 mg/kg, i.p. every other day). The CTRL group and the drug control groups were provided with standard rodent chow whereas the diseased groups received AD.

The survival analysis revealed that SMVT monotherapy (Figure 4A) resulted in a non-significant improvement in survival percent when compared to the DENA/TAA group. However, treatment with STA-9090 (Figure 4B) or the SMVT/STA-9090 combination (Figure 4C) significantly increased survival percentage compared to DENA/TAA.

### 3.4 STA/SMVT combination therapy alleviated liver function indicators in rats fed a HFD and exposed to DENA and TAA

**FIGURE 5:**
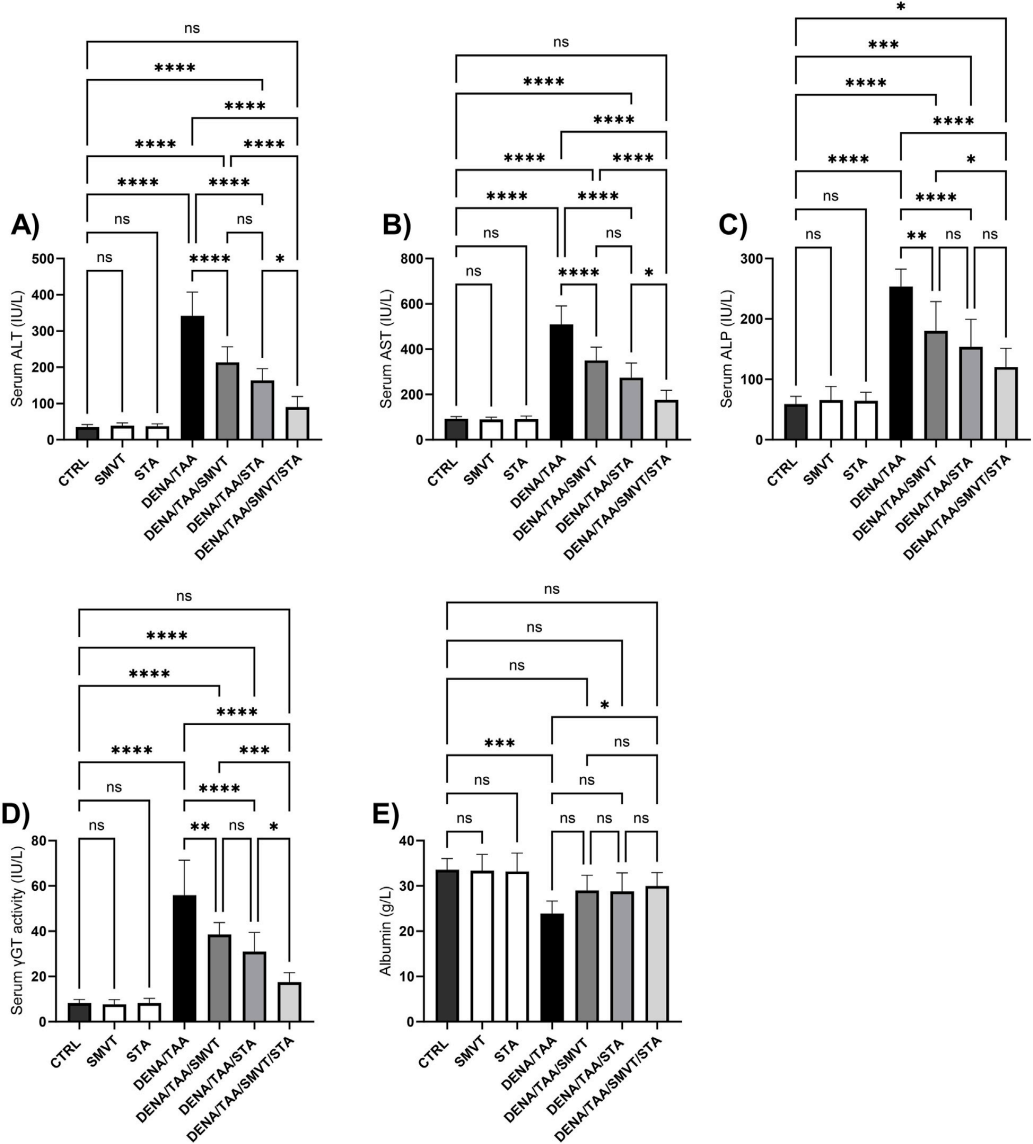
Effect of SMVT, STA-9090 (STA), and combination therapy on serum ALT **(A)**; AST **(B)**; ALP **(C)**; γ-GT **(D)**; and Albumin **(E)** in rats fed a HFD and exposed to DENA and TAA. Data are presented as the mean ± SD. ns, non-significant, *P < 0.05, **P < 0.01, ***P < 0.001, ****P < 0.0001. CTRL, normal control group received the vehicle; SMVT, normal group received SMVT (30 mg/kg/day); STA, normal group received STA-9090 (10 mg/kg, i.p. every other day); DENA/TAA, DENA/TAA-induced fibrosis group received the vehicle; DENA/TAA/SMVT, DENA/TAA-induced fibrosis group treated with SMVT (30 mg/kg/day); DENA/TAA/STA, DENA/TAA-induced fibrosis group treated with STA-9090 (10 mg/kg, i.p. every other day); DENA/TAA/STA/SMVT, DENA/TAA-induced fibrosis group treated with SMVT (30 mg/kg/day) and STA-9090 (10 mg/kg, i.p. every other day). The CTRL group and the drug control groups were provided with standard rodent chow whereas the diseased groups received AD.

Compared to the CTRL group, the DENA/TAA group showed significantly increased levels of ALT (Figure 5A), AST (Figure 5B), ALP (Figure 5C), and γGT (Figure 5D), as well as decreased levels of albumin (Figure 5E), indicating impaired liver function. SMVT, STA, or their combined administration resulted in a significant reduction in ALT, AST, ALP, and γGT levels in comparison to the DENA/TAA group. The combined treatment demonstrated the most potent effect among them. Furthermore, solely the combination therapy displayed a substantial elevation in serum albumin levels compared to the DENA/TAA group.

### 3.5 SMVT corrected dyslipidemia induced by multiple liver insults

**FIGURE 6:**
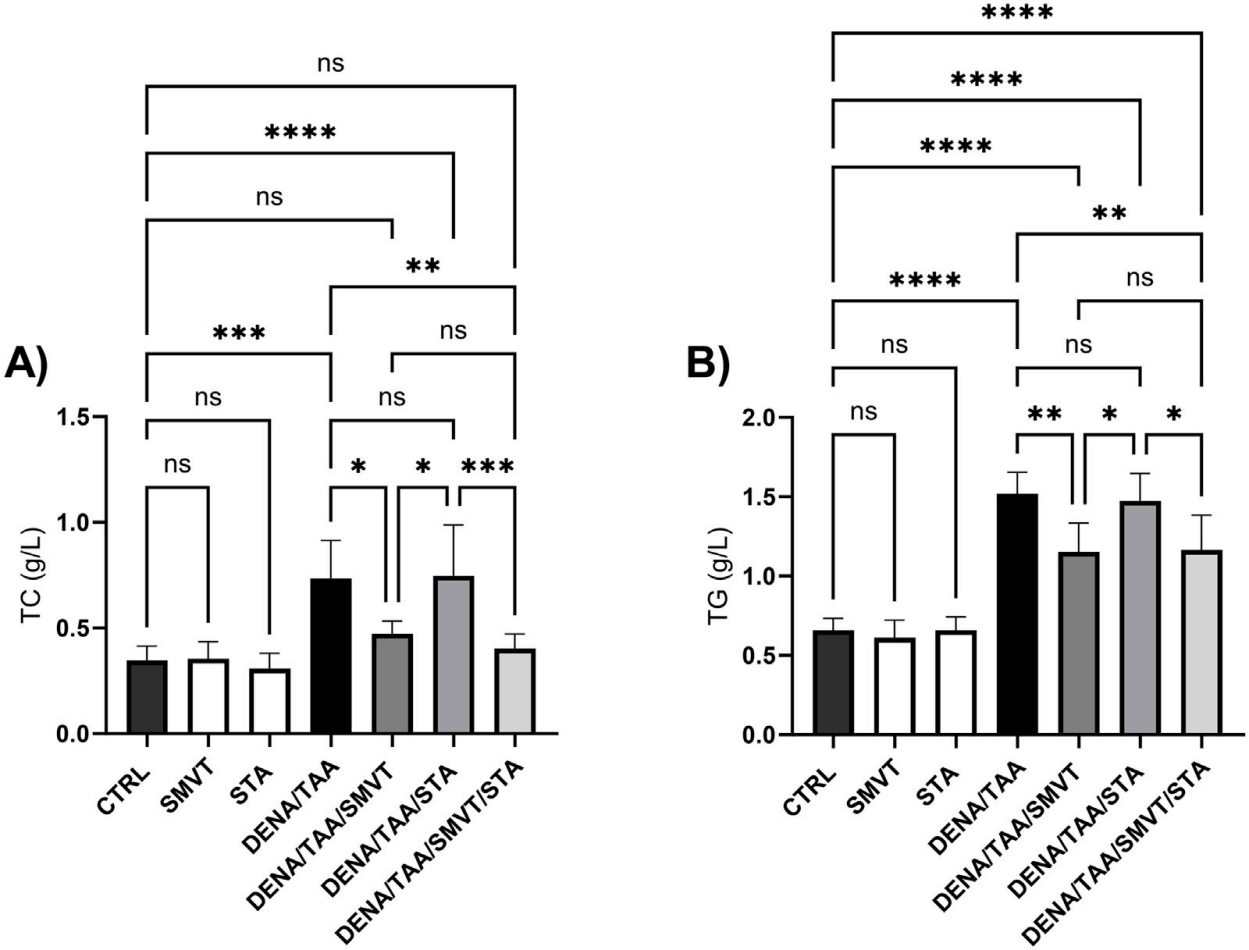
Effect of SMVT, STA-9090 (STA), and combination therapy on total cholesterol **(A)**; and triglyceride **(B)** in rats fed a HFD and exposed to DENA and TAA. Data are presented as the mean ± SD. ns, non-significant, *P < 0.05, **P < 0.01, ***P < 0.001, ****P < 0.0001. CTRL, normal control group received the vehicle; SMVT, normal group received SMVT (30 mg/kg/day); STA, normal group received STA-9090 (10 mg/kg, i.p. every other day); DENA/TAA, DENA/TAA-induced fibrosis group received the vehicle; DENA/TAA/SMVT, DENA/TAA-induced fibrosis group treated with SMVT (30 mg/kg/day); DENA/TAA/STA, DENA/TAA-induced fibrosis group treated with STA-9090 (10 mg/kg, i.p. every other day); DENA/TAA/STA/SMVT, DENA/TAA-induced fibrosis group treated with SMVT (30 mg/kg/day) and STA-9090 (10 mg/kg, i.p. every other day). The CTRL group and the drug control groups were provided with standard rodent chow whereas the diseased groups received AD.

The DENA/TAA group (a rat group that received AD) displayed significantly elevated TC (Figure 6A) and TG (Figure 6B) *versus* CTRL. SMVT treatment significantly lowered both TC and TG *versus* DENA/TAA. STA-9090 did not significantly alter TC or TG. The combination therapy significantly reduced TC and TG compared to DENA/TAA and showed greater decreases in these lipids than STA-9090 alone. An effect that is attributed to the SMVT treatment.

### 3.6 STA/SMVT combination therapy mitigated disturbances in liver histology induced by DENA/TAA and HFD in the rat liver

**FIGURE 7:**
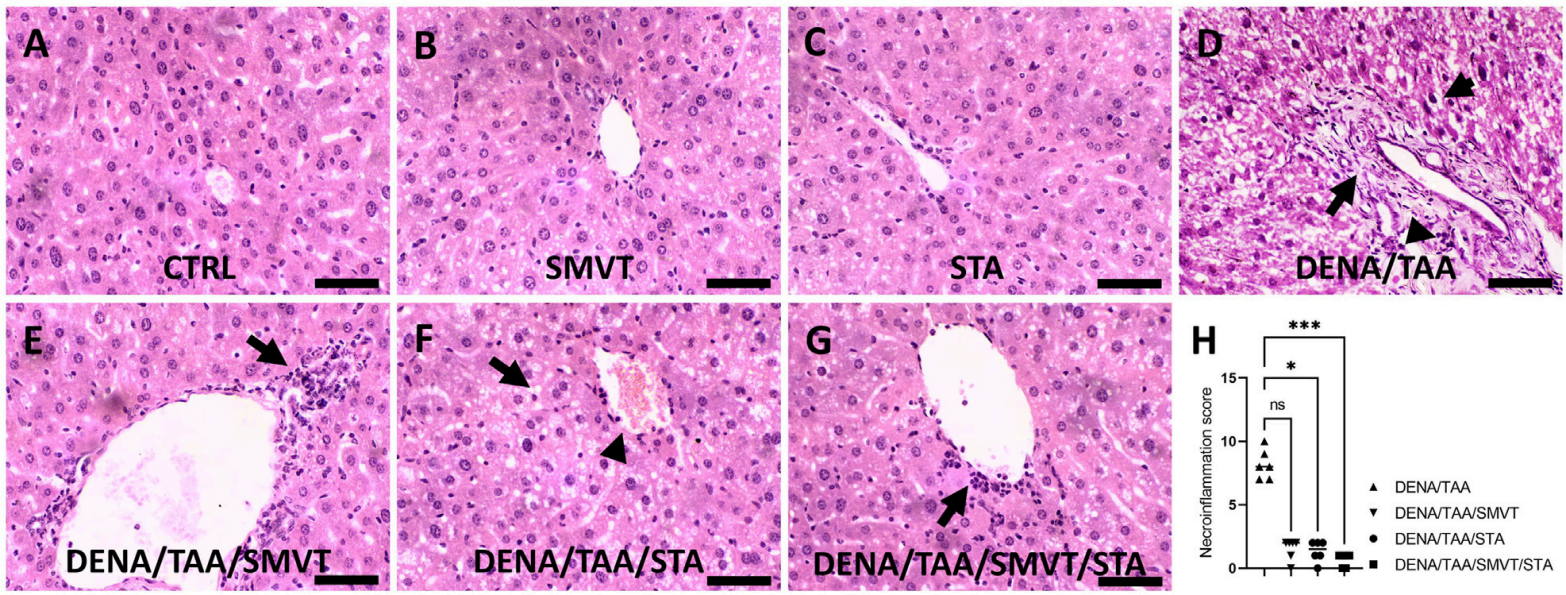
Effect of SMVT, STA-9090 (STA), and combination therapy on histopathological characteristics and inflammation score in rats fed a HFD and exposed to DENA and TAA. The figure presents photomicrographs of H&E-stained liver sections depicting hepatic architecture in various experimental groups. The CTRL **(A)**, SMVT **(B)**, and STA **(C)** groups exhibit normal hepatic structure, while the DENA/TAA group **(D)** displays disrupted architecture with fat droplet accumulation, hepatocyte ballooning (short arrow), fibrotic tissue deposition (long arrow), inflammatory cell infiltration (arrowhead), and cellular atypia. Treatment with SMVT **(E)** improves hepatic structure, attenuating fibrosis and fat accumulation with a residual inflammatory response (arrow). STA-9090 treatment **(F)** also improves hepatic structure, reducing fibrosis (arrowhead) and inflammation, but fat droplets persist (arrow). Combined SMVT/STA-9090 treatment **(G)** enhances hepatic structure, minimizing fibrosis and inflammation (arrow), and reducing fat droplets. Necro-inflammatory scores **(H)** confirm decreased inflammation with SMVT, STA, and SMVT/STA compared to DENA/TAA, with the combination therapy showing the lowest score, aligning with histological improvements. The scale bar represents 50 µm.

Photomicrographs of H&E-stained liver sections are shown in Figure 7. The CTRL (A), SMVT (B), and STA (C) groups displayed normal hepatic structure. In contrast, the DENA/TAA group (D) showed disturbed hepatic architecture showing fat droplet accumulation and hepatocyte ballooning (short arrow), fibrotic tissue deposition (long arrow), inflammatory cell infiltration (arrowhead), and cellular atypia (early dysplastic alterations). Treatment with SMVT (E) resulted in improved hepatic structure and attenuated fibrotic tissue deposition and fat droplet accumulation. However, a moderate level of inflammatory cell infiltration still exists (arrow). Treatment with STA-9090 (F) resulted in improved hepatic structure and attenuated fibrotic tissue deposition (arrowhead) and inflammatory cell infiltration. Nevertheless, fat droplet accumulation still exists (arrow). The combined treatment of SMVT/STA-9090 (G) exhibited enhanced hepatic structure, characterized by a predominantly absence of fibrotic tissue deposition and a mild degree of inflammatory cell infiltration (arrow), along with reduced fat droplet accumulation. The scale bar = 50 µm. Evaluation of necro-inflammatory scores (H) confirmed higher scores in the DENA/TAA group compared to CTRL. Treatments involving SMVT, STA-9090, and SMVT/STA-9090 significantly decreased necro-inflammatory scores compared to the DENA/TAA group. Notably, the combination therapy yielded the lowest necro-inflammatory score, aligning with observed histological improvements.

### 3.7 STA/SMVT combination therapy mitigated fibrotic tissue deposition induced by multiple insults in the rat liver

**FIGURE 8:**
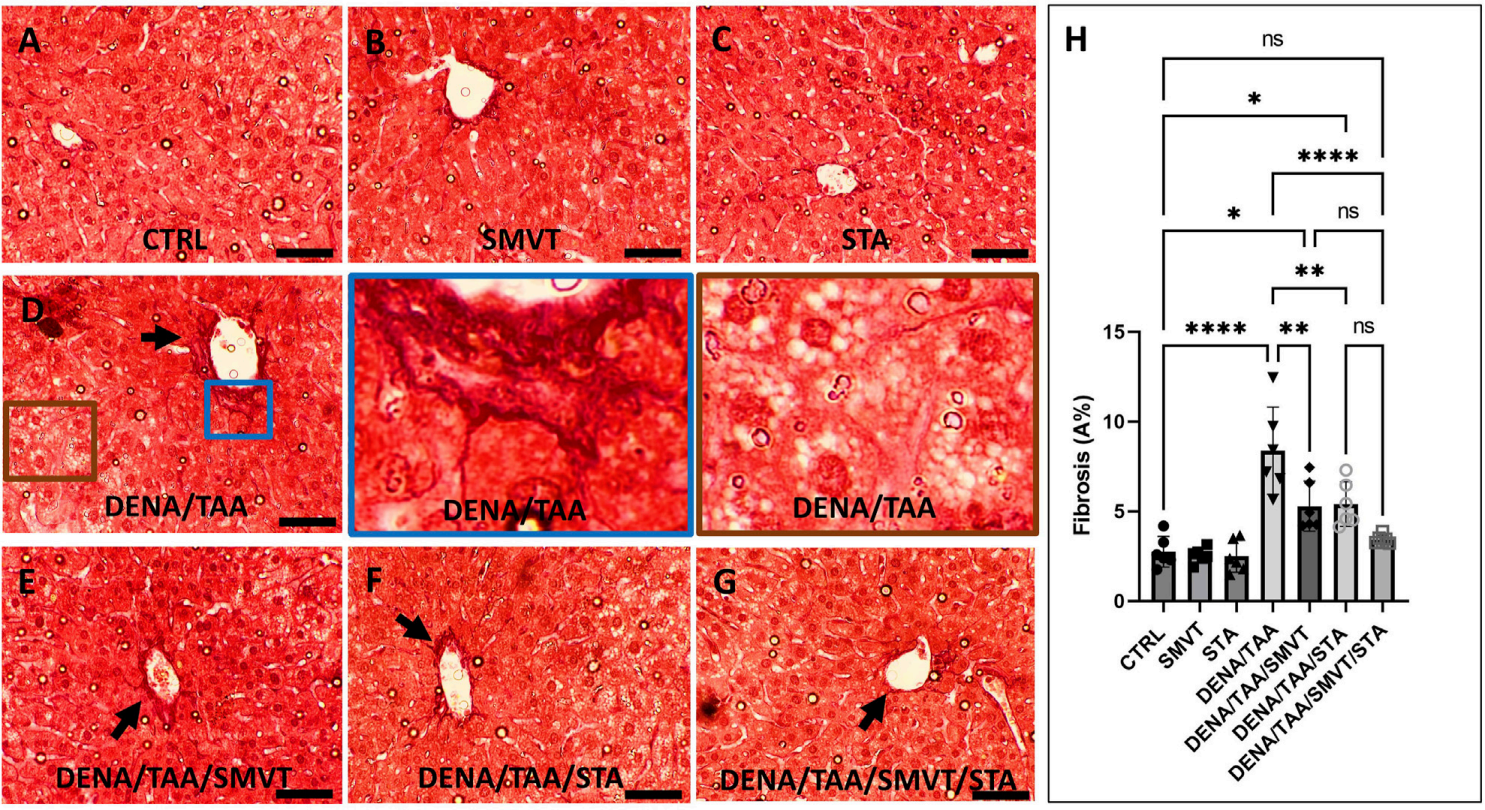
Effect of SMVT, STA-9090 (STA), and combination therapy on the degree of fibrotic tissue deposition in rats fed a HFD and exposed to DENA and TAA. In this figure, representative photomicrographs of Sirius red-stained liver sections are presented. The CTRL **(A)**, SMVT **(B)**, and STA **(C)** groups exhibit typical collagen deposition patterns, indicative of normal liver histology. Conversely, the DENA/TAA group **(D)** displays an evident increase in fibrotic tissue deposition (arrow). Treatment with SMVT **(E)** and STA **(F)** individually results in a noticeable reduction in fibrotic tissue deposition (arrows). Remarkably, the combined treatment with SMVT/STA **(G)** leads to a further reduction in fibrotic tissue deposition, resembling a pattern more or less similar to the normal group (arrow). The scale bar is set at 50 µm for reference. Fibrosis scores **(H)** confirm higher scores in the DENA/TAA group compared to CTRL, while treatments involving SMVT, STA-9090, and SMVT/STA-9090 significantly decrease fibrosis scores in comparison to DENA/TAA. Notably, the combination therapy yields the lowest fibrosis score among the treatments, underscoring its efficacy in mitigating liver fibrosis.

Figure 8 displays photomicrographs of Sirius red-stained liver sections. The CTRL (A), SMVT (B), and STA (C) groups exhibited normal collagen deposition. In contrast, the DENA/TAA group (D) displayed increased fibrotic tissue deposition (arrow). Treatment with SMVT (E) and STA-9090 (F) resulted in reduced fibrotic tissue deposition (arrow). The combined treatment with SMVT/STA-9090 (G) led to a further reduction in fibrotic tissue deposition, resembling a pattern more or less similar to normal (arrow). The scale bar = 50 µm. Evaluation of fibrosis area % (H) verified higher % in the DENA/TAA group compared to CTRL. Treatments involving SMVT, STA-9090, and SMVT/STA-9090 significantly decreased fibrosis area in comparison to DENA/TAA, with the combination therapy yielding the lowest fibrosis area %.

### 3.8 STA/SMVT combination therapy decreased tissue expression of α-SMA

**FIGURE 9:**
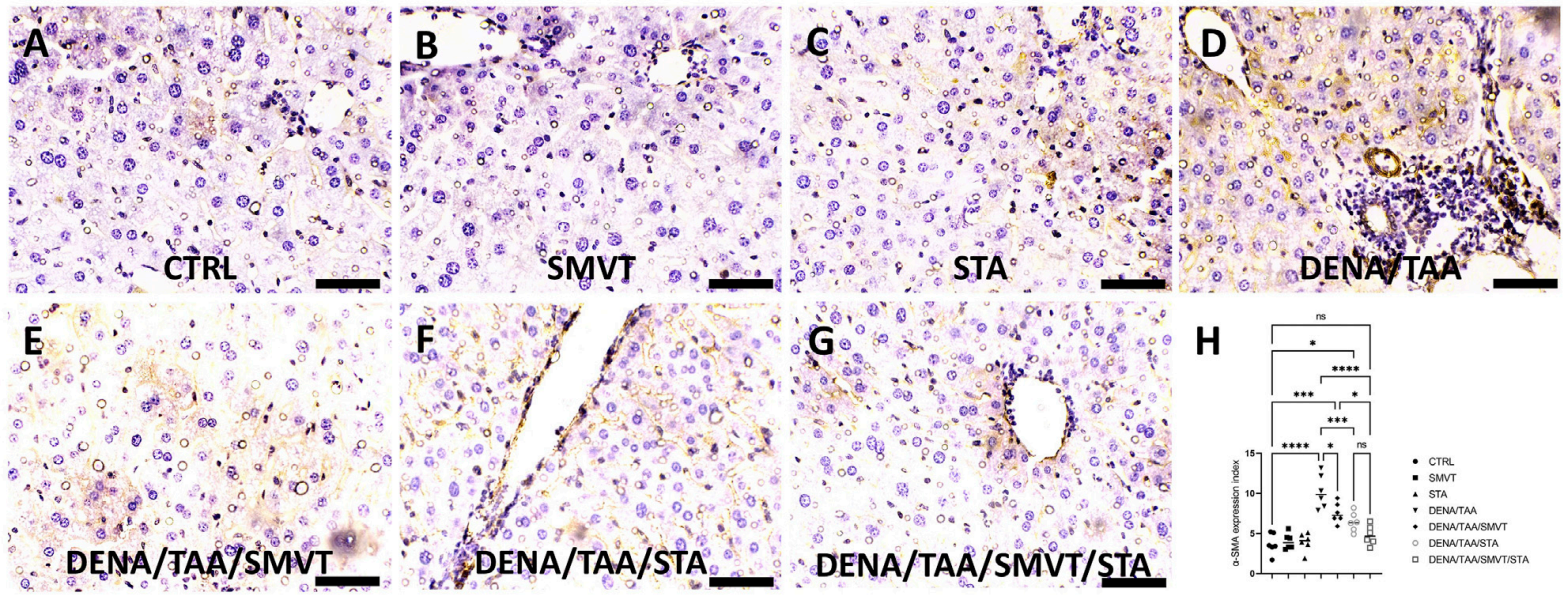
Effect of SMVT, STA-9090 (STA), and combination therapy on the tissue expression of α-SMA in rats fed a HFD and exposed to DENA and TAA. In this figure, α-SMA-immunostained liver sections are depicted, revealing distinctive tissue expression patterns. The CTRL **(A)**, SMVT **(B)**, and STA **(C)** groups display a normal level of tissue expression. Conversely, the DENA/TAA group **(D)** exhibits heightened tissue expression (arrow). Treatment with SMVT **(E)** and STA **(F)** individually results in a reduction of tissue expression (arrow). Impressively, the combined treatment with SMVT/STA **(G)** further diminishes tissue expression, resembling a pattern more or less similar to the normal group (arrow). The scale bar is set at 50 µm for reference. The α-SMA expression index **(H)** verifies a higher level in the DENA/TAA group compared to CTRL. In contrast, treatments involving SMVT, STA-9090, and SMVT/STA-9090 significantly decrease the expression indices compared to DENA/TAA. Notably, the combination therapy achieves the lowest expression index, underscoring its effectiveness in mitigating α-SMA-associated liver pathology.

In Figure 9, photomicrographs of α-SMA-immunostained liver sections are presented. The CTRL (A), SMVT (B), and STA (C) groups demonstrated normal tissue expression. Conversely, the DENA/TAA group (D) exhibited heightened tissue expression (indicated by the arrow). Treatment with SMVT (E) and STA-9090 (F) resulted in a reduction of tissue expression (arrow). The combined treatment with SMVT/STA-9090 (G) further decreased tissue expression, resembling a pattern more or less similar to normal (arrow). The scale bar is 50 µm. Evaluation of the α-SMA expression index (H) confirmed higher indices in the DENA/TAA group compared to CTRL. Treatments involving SMVT, STA-9090, and SMVT/STA-9090 significantly decreased indices compared to DENA/TAA, with the combination therapy achieving the lowest expression index.

### 3.9 STA/SMVT combination decreased TNF-α and MPO activity and did not enhance oxidative stress reduction beyond what is achieved by SMVT alone

**FIGURE 10:**
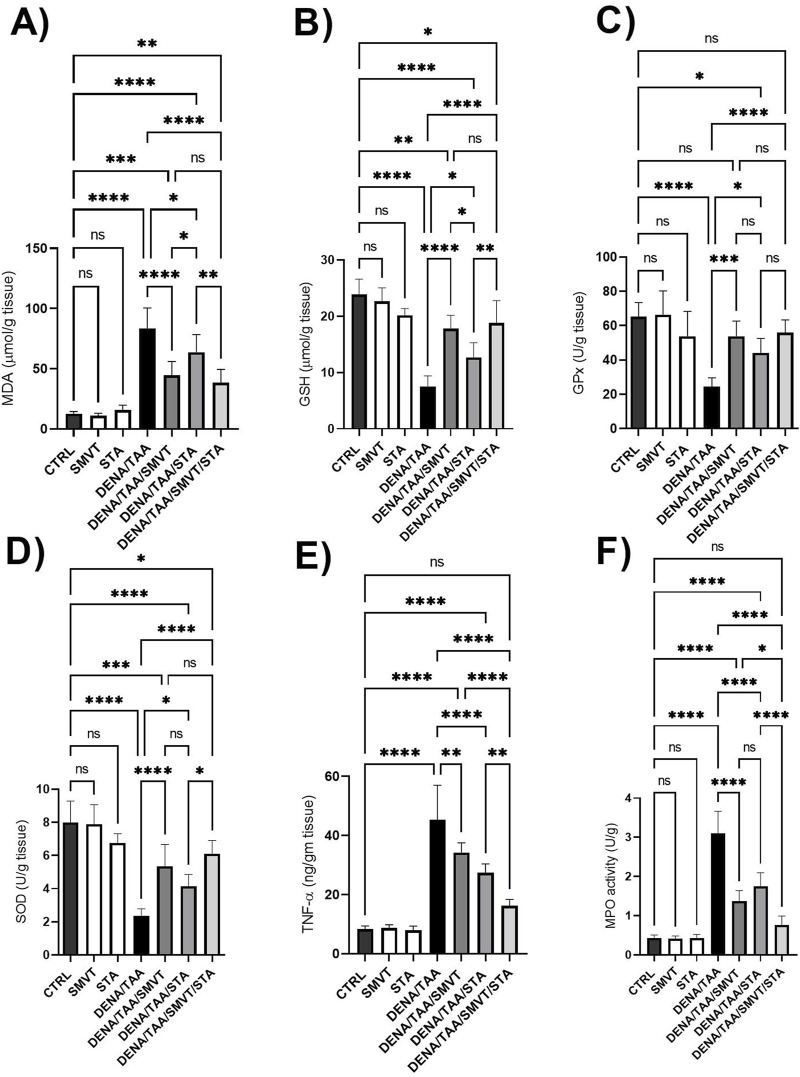
Effect SMVT, STA-9090 (STA), and combination therapy on MDA **(A)**; GSH **(B)**; GPx **(C)**; SOD **(D)**; TNF-α **(E)**, and MPO **(F)** in rats fed a HFD and exposed to DENA and TAA. Data are presented as the mean ± SD. ns, non-significant, *P < 0.05, **P < 0.01, ***P < 0.001, ****P < 0.0001. CTRL, normal control group received the vehicle; SMVT, normal group received SMVT (30 mg/kg/day); STA, normal group received STA-9090 (10 mg/kg, i.p. every other day); DENA/TAA, DENA/TAA-induced fibrosis group received the vehicle; DENA/TAA/SMVT, DENA/TAA-induced fibrosis group treated with SMVT (30 mg/kg/day); DENA/TAA/STA, DENA/TAA-induced fibrosis group treated with STA-9090 (10 mg/kg, i.p. every other day); DENA/TAA/STA/SMVT, DENA/TAA-induced fibrosis group treated with SMVT (30 mg/kg/day) and STA-9090 (10 mg/kg, i.p. every other day). The CTRL group and the drug control groups were provided with standard rodent chow whereas the diseased groups received AD.

Compared to CTRL, the DENA/TAA group showed significantly increased MDA (Figure 10A) and decreased GSH (Figure 10B), GPx (Figure 10C), and SOD (Figure 10D), indicating heightened oxidative stress. Both SMVT and STA-9090 significantly improved MDA, GSH, GPx, and SOD compared to DENA/TAA. The combination therapy significantly decreased MDA and increased GSH, GPx, and SOD compared to DENA/TAA, and demonstrated greater antioxidant effects than STA; while showing no significant difference when compared to SMVT. Additionally, compared to CTRL, the DENA/TAA group showed elevated TNF-α and MPO (Figures 10E, F, respectively) indicating heightened inflammation and neutrophil infiltration. Both SMVT and STA-9090 significantly lowered TNF-α and MPO compared to DENA/TAA. The combination therapy exhibited a greater reduction in TNF-α levels compared to either of the monotherapies indicating reduced inflammation and neutrophil infiltration in hepatic tissue.

### 3.10 Effect on fibrosis markers

**FIGURE 11:**
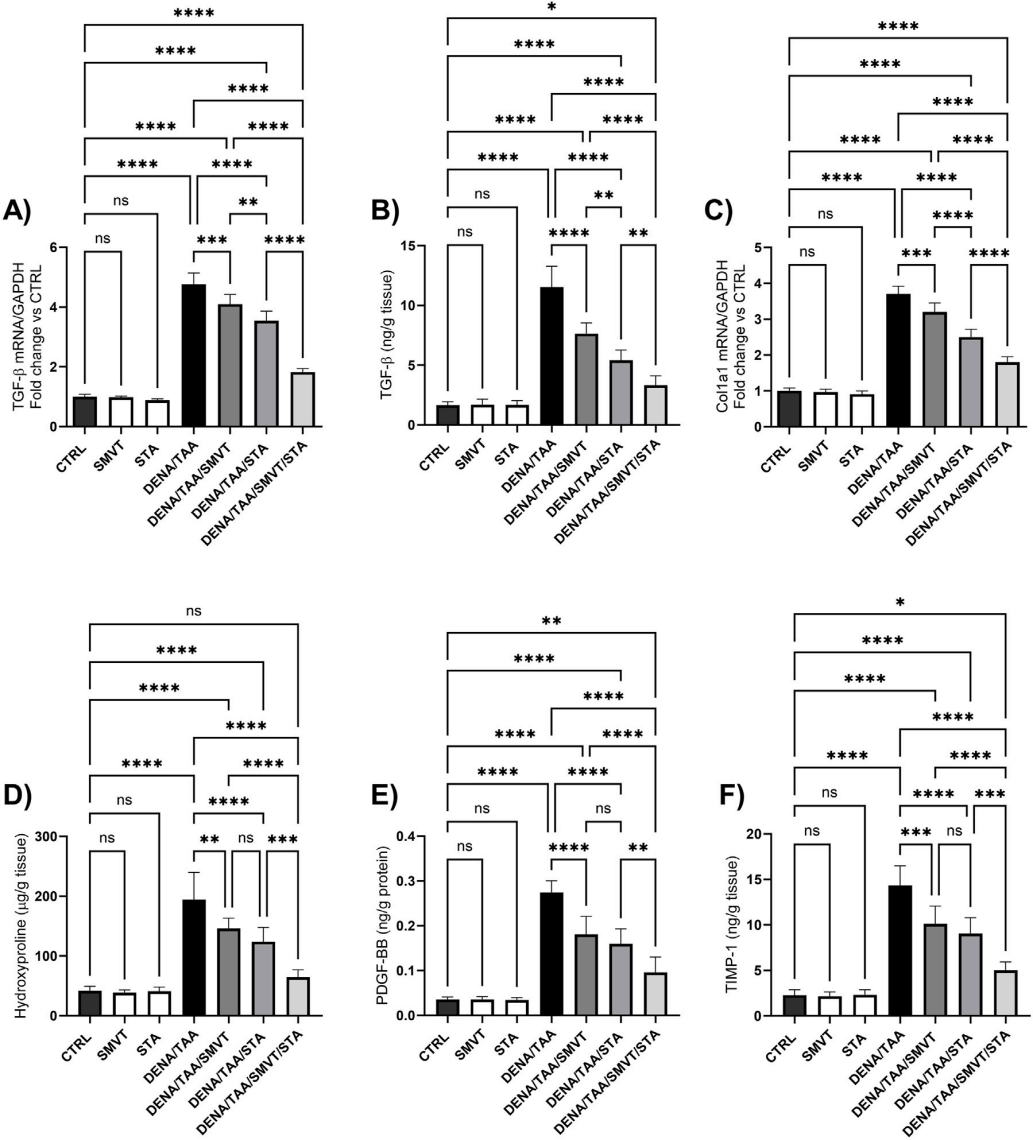
Effect of SMVT, STA-9090 (STA), and combination therapy on TGF-β mRNA **(A)**; TGF-β protein **(B)**; Col1a1 mRNA **(C)**; Hydroxyproline **(D)**; PDGF-BB **(E)**; and TIMP-1 **(F)** in rats fed a HFD and exposed to DENA and TAA. Data are presented as the mean ± SD. ns, non-significant, *P < 0.05, **P < 0.01, ***P < 0.001, ****P < 0.0001. CTRL, normal control group received the vehicle; SMVT, normal group received SMVT (30 mg/kg/day); STA, normal group received STA-9090 (10 mg/kg, i.p. every other day); DENA/TAA, DENA/TAA-induced fibrosis group received the vehicle; DENA/TAA/SMVT, DENA/TAA-induced fibrosis group treated with SMVT (30 mg/kg/day); DENA/TAA/STA, DENA/TAA-induced fibrosis group treated with STA-9090 (10 mg/kg, i.p. every other day); DENA/TAA/STA/SMVT, DENA/TAA-induced fibrosis group treated with SMVT (30 mg/kg/day) and STA-9090 (10 mg/kg, i.p. every other day). The CTRL group and the drug control groups were provided with standard rodent chow whereas the diseased groups received AD.

The DENA/TAA group displayed marked increases in the levels of TGF-β mRNA and protein (Figures 11A, B, respectively), Col1a1 mRNA (Figure 11C), hydroxyproline (Figure 11D), PDGF-BB (Figure 11E) and TIMP-1 (Figure 11F) compared to CTRL, suggestive of developed tissue fibrosis. Both SMVT and STA-9090 monotherapies significantly reduced all these markers *versus* DENA/TAA. The combination treatment decreased these fibrosis markers to a greater extent than either monotherapy. STA-9090 resulted in a significantly decreased TGF-β (mRNA and protein) and Col1a1 mRNA when compared to SMVT.

### 3.11 Effect on hedgehog signaling pathway

**FIGURE 12:**
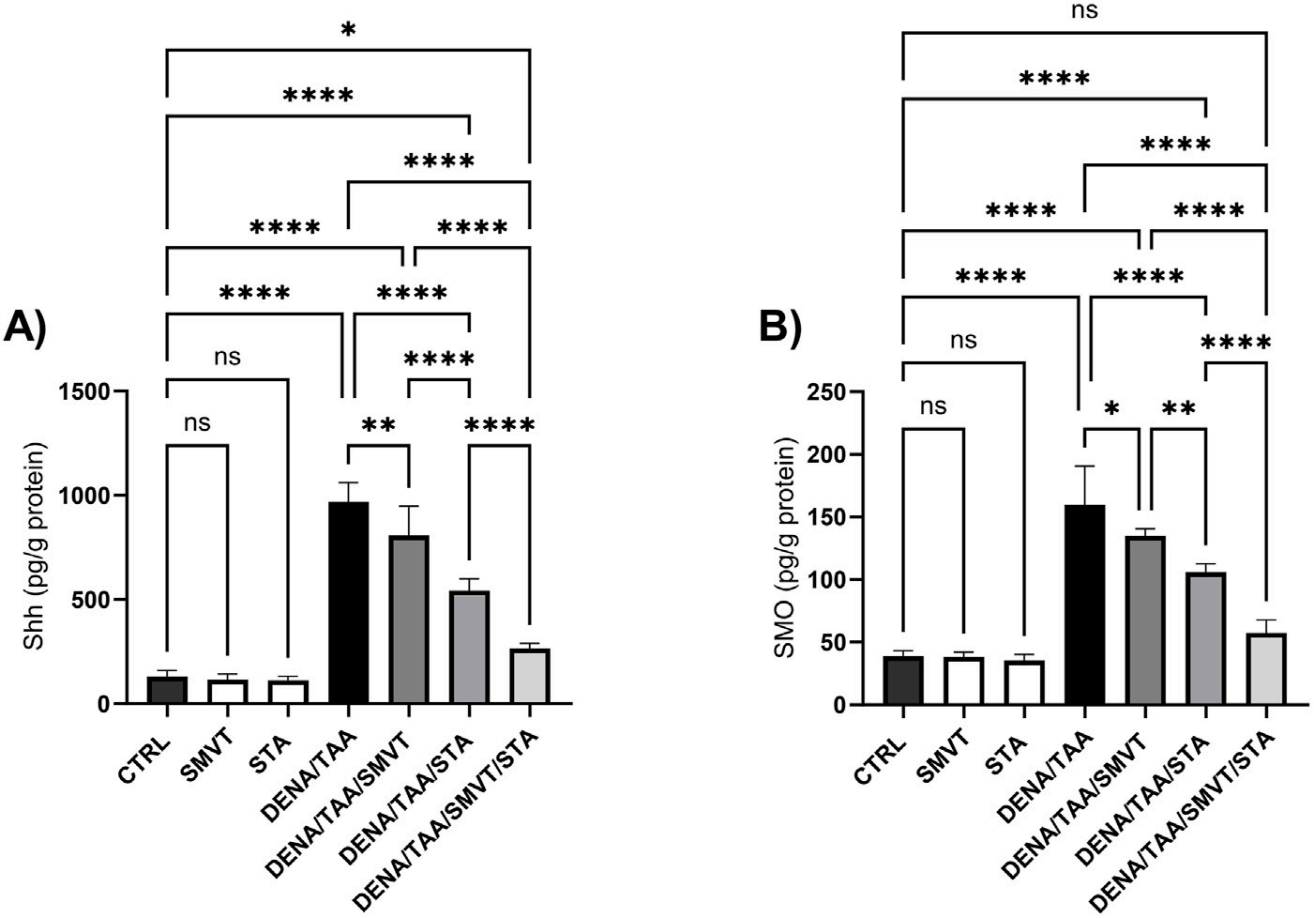
Effect of SMVT, STA-9090 (STA), and combination therapy on Shh **(A)**; and SMO **(B)** in rats fed a HFD and exposed to DENA and TAA. Data are presented as the mean ± SD. ns, non-significant, *P < 0.05, **P < 0.01, ***P < 0.001, ****P < 0.0001. CTRL, normal control group received the vehicle; SMVT, normal group received SMVT (30 mg/kg/day); STA, normal group received STA-9090 (10 mg/kg, i.p. every other day); DENA/TAA, DENA/TAA-induced fibrosis group received the vehicle; DENA/TAA/SMVT, DENA/TAA-induced fibrosis group treated with SMVT (30 mg/kg/day); DENA/TAA/STA, DENA/TAA-induced fibrosis group treated with STA-9090 (10 mg/kg, i.p. every other day); DENA/TAA/STA/SMVT, DENA/TAA-induced fibrosis group treated with SMVT (30 mg/kg/day) and STA-9090 (10 mg/kg, i.p. every other day). The CTRL group and the drug control groups were provided with standard rodent chow whereas the diseased groups received AD.

**FIGURE 13:**
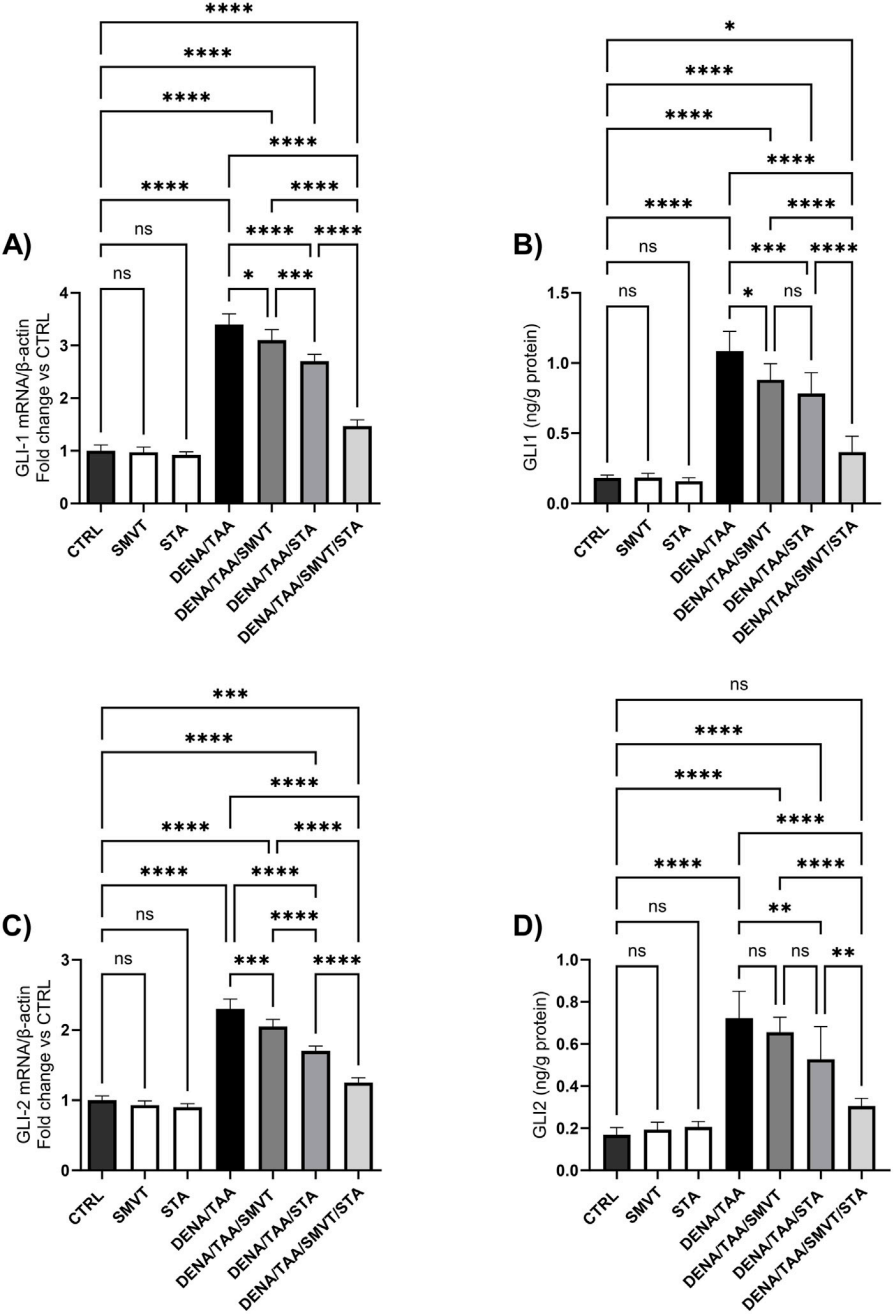
Effect of SMVT, STA-9090 (STA), and combination therapy on GLI-1 mRNA **(A)**; GLI-1 protein **(B)**; GLI-2 mRNA **(C)**; and GLI-2 protein **(D)** in rats fed a HFD and exposed to DENA and TAA. Data are presented as the mean ± SD. ns, non-significant, *P < 0.05, **P < 0.01, ***P < 0.001, ****P < 0.0001. CTRL, normal control group received the vehicle; SMVT, normal group received SMVT (30 mg/kg/day); STA, normal group received STA-9090 (10 mg/kg, i.p. every other day); DENA/TAA, DENA/TAA-induced fibrosis group received the vehicle; DENA/TAA/SMVT, DENA/TAA-induced fibrosis group treated with SMVT (30 mg/kg/day); DENA/TAA/STA, DENA/TAA-induced fibrosis group treated with STA-9090 (10 mg/kg, i.p. every other day); DENA/TAA/STA/SMVT, DENA/TAA-induced fibrosis group treated with SMVT (30 mg/kg/day) and STA-9090 (10 mg/kg, i.p. every other day). The CTRL group and the drug control groups were provided with standard rodent chow whereas the diseased groups received AD.

The DENA/TAA group resulted in significantly increased Shh (Figure 12A), SMO (Figure 12B), GLI1 mRNA/protein (Figures 13A, B), and GLI2 mRNA/protein (Figures 13C, D) *versus* CTRL, suggestive of Hedgehog pathway activation. STA-9090 significantly reduced Shh, SMO, GLI1 mRNA/protein, and GLI2 mRNA/protein compared to DENA/TAA. SMVT significantly lowered Shh, GLI1 mRNA/protein, and GLI2 mRNA but not GLI2 protein (however a considerable reduction is observed). The combination therapy decreased Shh, SMO, GLI1 mRNA/protein, and GLI2 mRNA/protein greater than either monotherapy.

### 3.12 Effect on heat shock proteins

**FIGURE 14:**
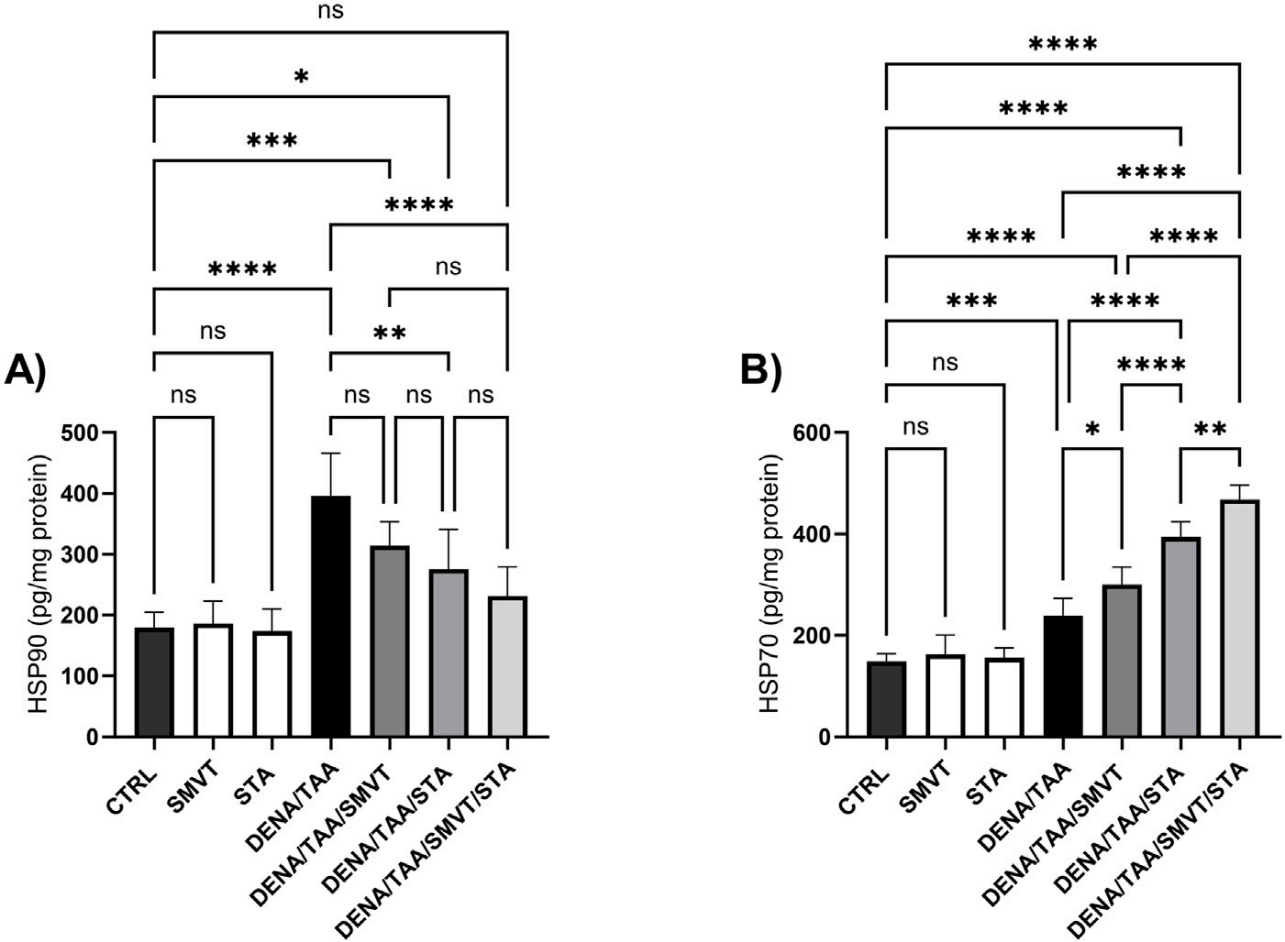
Effect of SMVT, STA-9090 (STA), and combination therapy on HSP90 **(A)**; and HSP70 **(B)** in rats fed a HFD and exposed to DENA and TAA. Data are presented as the mean ± SD. ns, non-significant, *P < 0.05, **P < 0.01, ***P < 0.001, ****P < 0.0001. CTRL, normal control group received the vehicle; SMVT, normal group received SMVT (30 mg/kg/day); STA, normal group received STA-9090 (10 mg/kg, i.p. every other day); DENA/TAA, DENA/TAA-induced fibrosis group received the vehicle; DENA/TAA/SMVT, DENA/TAA-induced fibrosis group treated with SMVT (30 mg/kg/day); DENA/TAA/STA, DENA/TAA-induced fibrosis group treated with STA-9090 (10 mg/kg, i.p. every other day); DENA/TAA/STA/SMVT, DENA/TAA-induced fibrosis group treated with SMVT (30 mg/kg/day) and STA-9090 (10 mg/kg, i.p. every other day). The CTRL group and the drug control groups were provided with standard rodent chow whereas the diseased groups received AD.

Compared to CTRL, the DENA/TAA group showed elevated HSP90 (Figure 14A) and HSP70 (Figure 14B). SMVT did not significantly alter HSP90 but increased HSP70. Both STA-9090 and SMVT/STA-9090 combined therapy significantly decreased HSP90 and increased HSP70 *versus* DENA/TAA.

## 4 Discussion

Liver fibrosis is a common consequence of chronic liver injury, progressing to cirrhosis and associated complications such as HCC (Lotersztajn et al., 2005). Early liver fibrosis has been suggested to be reversible. However, effective treatments are currently lacking (Tan et al., 2021). Consequently, the development of anti-fibrosis drugs remains a top research priority. Various strategies have emerged as crucial means to inhibit the occurrence and progression of liver fibrosis and associated dysplasia in recent years. In this study, we evaluated the individual and combined effects of SMVT and the HSP90 inhibitor, STA-9090, in a DENA/TAA rat model fed a HFD. This model was found to recapitulate key features of human liver fibrosis pathology in the settings of fatty and early dysplastic changes (Henderson et al., 2018). The current investigation provides evidence that the co-inhibition of HMG CoA reductase and HSP90 produces enhanced antifibrotic activity. Our findings suggest that the observed hepatoprotective role is linked to the inhibition of aberrant hedgehog signaling.

Administration of DENA/TAA/HFD significantly decreased the animals’ body weight by week 8 and continued to decrease till the end of week 16 when compared to control. The gradual body weight loss reflects declining nutritional status and cachexia associated with advanced liver disease (Stirnimann and Stirnimann, 2019; Wu et al., 2023). The ability of combination therapy (SMVT/STA) to attenuate this weight loss may indicate a maintenance of overall health. Also, the significant increase in the liver index may indicate hepatomegaly in DENA/TAA rats which aligns with chronic hepatic injury, while treatment groups show reduction towards normal liver weight. Additionally, the present investigation revealed a decreased survival proportion in the diseased group. The observed decline in the survival of rats subjected to DENA/TAA/HFD is indicative of the systemic complications associated with liver failure arising from chronic injury. Notably, the survival proportion witnessed a significant improvement with the administration of STA. Moreover, the combination treatment exhibited a prolonged lifespan, underscoring its effectiveness in slowing the progression of fibrosis. This outcome highlights the potential therapeutic benefits of the STA-9090 and the combination treatment (SMVT/STA) in mitigating the adverse effects on survival linked to chronic liver injury and fibrosis progression.

Administration of DENA/TAA and HFD in rats exhibited marked elevations in serum ALT, AST, ALP, and γ-GT signifying extensive hepatocellular damage. Reduction of these enzymes by treatments signifies attenuation of liver injury. In addition, decreased albumin relates to synthetic dysfunction, corrected only by combination therapy. Overall, monotherapies improved liver function, further enhanced by combination treatment. On the other hand, the buildup of lipids within hepatocytes is a known precursor to liver damage, instigating a cascade of events including inflammation, fibrosis, and cirrhosis. Beyond the presence of fatty acids and triglycerides, studies indicate an augmented accumulation of free cholesterol in the liver, exerting toxic effects and further contributing to hepatic injury (Malhotra et al., 2020). The accumulation of lipids in hepatocytes, stemming from conditions like dyslipidemia, results in liver damage and initiates a complex response leading to hepatic inflammation and fibrosis (Chatrath et al., 2012). Dyslipidemia is common in chronic liver disease due to impaired lipid metabolism (Beaven and Tontonoz, 2006). In the current study, an abnormal increase was found in serum TC and TG in response to DENA/TAA/AD. SMVT corrected the abnormally elevated TC and TG by inhibiting cholesterol synthesis. STA-9090 did not provide improvement, consistent with its lack of lipid-lowering effects.

Our observations suggest a complex interplay rather than a simple cause-effect relationship. The accumulation of lipids appears to be an early event in our model, preceding the development of significant fibrosis. This is evidenced by the onset of elevated serum TC and TG levels, which aligns with previous studies indicating that lipid accumulation can initiate a cascade of hepatocellular injury (Malhotra et al., 2020), and lipotoxicity may be an initiating factor in liver injury (Wiering et al., 2023). However, as fibrosis progresses, it likely exacerbates lipid dysregulation through impaired hepatic function, creating a feed-forward loop (Horn and Tacke, 2024). This bidirectional relationship between dyslipidemia and liver damage underscores the complexity of the pathogenic process and highlights the importance of early intervention strategies targeting both lipid metabolism and fibrogenic pathways.

The efficacy of SMVT in correcting dyslipidemia, coupled with the antifibrotic effects of both SMVT and STA-9090, supports a therapeutic approach that addresses both the initiating factors (lipid accumulation) and the consequent pathological processes (fibrosis) in liver disease. This multitarget approach aligns with recent findings demonstrating that interventions targeting lipid metabolism can attenuate fibrosis progression in experimental models (Summer and Mora, 2019; Chen et al., 2022; Zhao et al., 2022).

ROS and products of lipid peroxidation play demonstrable roles in key events of hepatic fibrogenesis, such as the activation and effects of stellate cells, as well as the expression of specific inhibitors of metalloproteinases. Reactive oxidant species are likely contributors to both the onset and progression of fibrosis. Oxidative stress and inflammation are key drivers of fibrogenesis (Kisseleva and Brenner, 2021). Both SMVT and STA-9090 attenuated these pathways based on reduced TNF-α and improved antioxidant status as well as decreased neutrophil infiltration as evidenced by lower activity of MPO. Combination therapy had superior effects, likely contributing to its enhanced antifibrotic action. Furthermore, histological analysis confirmed combination therapy produced near-normal liver histology with minimal fibrosis. Microscopic evidence of liver injury and collagen deposition in DENA/TAA rats fed an HFD was mitigated by treatments, confirming their hepatoprotective and anti-fibrotic effects on tissue level. Combination therapy produced near-normal histology, consistent with optimal antifibrotic activity.

Elimination of myeloid TGF-β through genetic deletion or inhibition of TGF-β through pharmacological means has been documented to alleviate liver fibrosis in murine models. In contrast, the induction of fibrosis in targeted tissues and organs, including the liver, occurs spontaneously with genetic overexpression of TGF-β. This underscores the critical role of TGF-β as a central mediator in the fibrotic process (Seki et al., 2007; Krenkel and Tacke, 2017). A significant upregulation of TGF-β, Col1a1, hydroxyproline, PDGF, and TIMP-1 observed in DENA/TAA/AD-treated rats signifies activation of fibrogenic pathways. Treatments suppressed these markers, corroborating their antifibrotic effects. Combination therapy decreased fibrosis markers further than individual drugs, underpinning its efficacy.

The hedgehog pathway is vital for regulating tissue polarity and stem cell populations. A key signal transducer in this pathway is the seven-transmembrane protein SMO, whose activity is controlled by the transmembrane protein Patched 1. When an active hedgehog ligand as Shh binds to the Patched one receptor, it relieves the inhibition on SMO, enabling downstream signaling events that culminate in the activation of GLI transcription factors. The GLI proteins act as transcription factors that can bind to specific gene promoter regions and thereby regulate the expression of target genes (Sasaki et al., 1997; Hu et al., 2015). HSP90, a molecular chaperone, plays a pivotal role in the activation of numerous signaling proteins within eukaryotic cells, among them the Shh pathway (Pearl and Prodromou, 2006). Increased Shh, SMO, GLI1, and GLI2, as well as, HSPs expression was found in DENA/TAA rats fed an HFD indicating aberrant hedgehog activation in the present investigation. Hedgehog signaling must be precisely controlled in adult liver cells to maintain liver health. Aberrant activation of hedgehog signaling has been implicated in various human diseases, including cancer and fibrosis. Excessive hedgehog pathway activity has been identified in these pathological conditions, contributing to disease progression (Yauch et al., 2009; Hu et al., 2015; Kramann, 2016). Therefore, targeting the hedgehog cascade may represent a therapeutic strategy for diseases involving abnormal stimulation of this important regulatory pathway.

STA-9090 significantly inhibited Shh, SMO, GLI1, GLI2, and HSP90 and elevated HSP70. Inhibition of HSP90 by STA-9090 results in the accumulation of unfolded or misfolded proteins within cells. This activates the heat shock response pathway, leading to upregulated expression of HSP70. HSP70 plays a vital role in refolding or degrading the misfolded proteins that arise due to impaired HSP90 function. There is evident collaboration between HSP90 and HSP70, and inhibiting HSP90 places a greater workload on HSP70 to handle protein folding and prevent aggregation (Taniguchi et al., 2014; Tsai et al., 2016). To compensate for the loss of HSP90 activity, the cell boosts HSP70 levels. Therefore, increased HSP70 expression serves as a marker of effective HSP90 inhibition (Li et al., 2020).

The elevated hedgehog signaling components (Shh, SMO, GLI1, and GLI2) observed in this study were significantly reduced by treatment with SMVT. This can be attributed to SMVT’s ability to inhibit cholesterol synthesis. Cholesterol can influence hedgehog signaling by modifying the conformation of the Patched one receptor. Additionally, cholesterol biosynthesis is necessary for the activity of the SMO protein in the hedgehog cascade (Gordon et al., 2018). Furthermore, the binding of cholesterol to the extracellular domain of SMO enables it to modulate interactions with Patched receptors (Hu and Song, 2019). When cholesterol levels are very low, inadequate membrane cholesterol may prevent Hedgehog from eliciting full signaling (Brown, 2007). The signaling capacity of Shh depends on its cholesterol modification at the N-terminus by the enzyme Hedgehog acyltransferase (Tuladhar et al., 2019). By inhibiting HMG-CoA reductase and lowering cellular cholesterol, SMVT indirectly blocks this cholesterol modification of Shh by Hedgehog acyltransferase. This results in impaired Shh signaling.

The current study offers several strengths that contribute to its significance in the field of hepatoprotective research. Firstly, our utilization of the DENA/TAA rat model fed a HFD provides a robust platform for investigating liver fibrosis in the context of fatty liver and early dysplastic changes. Secondly, our comprehensive approach, encompassing biochemical, histological, and molecular analyses, offers a multifaceted view of the hepatoprotective effects of SMVT and STA-9090, both individually and in combination. The demonstration of enhanced efficacy with combination therapy highlights the potential of multi-target approaches in managing complex liver pathologies. However, it is important to acknowledge certain limitations of our study. The use of a single animal model, while relevant, may not fully capture the heterogeneity of human liver diseases. Future studies incorporating additional models or human liver organoids could further validate our findings. Additionally, while our study provides insights into the mechanisms of action, particularly regarding the Hedgehog pathway, a more in-depth exploration of other potential pathways affected by the combination therapy could yield additional valuable insights. A useful future approach would be to use a downstream enhancer of the pathway in combination with the different treatments to better elucidate the specific role of the pathway.

## 5 Conclusion

In conclusion, this study demonstrates the therapeutic potential of targeting aberrant hedgehog signaling using SMVT and the HSP90 inhibitor STA-9090, alone and in combination, for the management of liver fibrosis. The DENA/TAA rat model fed an HFD effectively recapitulates key features of human fibrosis pathology, including elevated hedgehog pathway activity. SMVT suppressed hedgehog signaling by reducing cholesterol biosynthesis required for Hedgehog ligand function. In parallel, STA-9090 directly inhibited the HSP90 chaperone essential for Hedgehog pathway activity. Using these drugs with complementary mechanisms allowed dual targeting of the hedgehog cascade. Monotherapies with SMVT or STA-9090 alleviated liver injury, inflammation, oxidative stress, tissue damage, and hedgehog marker expression. Combination therapy provided further significant improvements in these parameters, including near-normal liver histology. Furthermore, our *in vitro* experiments unveiled a synergistic cytotoxic impact on TGF-β-treated HepG2 cells. These cells were found to exhibit EMT characteristics and display a fibroblast-like morphology. Collectively, SMVT and STA-9090 exhibited complementary antifibrotic mechanisms targeting cholesterol and HSP90 activity, respectively. Their combined use produces enhanced therapeutic effects in livers exposed to multiple insults, supporting further investigation of this promising approach.

## Data Availability

The original contributions presented in the study are included in the article/supplementary material, further inquiries can be directed to the corresponding authors.

## Abbreviations

ALP, Alkaline phosphatase; ALT, Alanine aminotransferase; AST, Aspartate aminotransferase; Col1A1, Collagen type I alpha 1; CTRL, Control; DENA, Diethyl nitrosamine; ECM, Extracellular matrix; EMT, epithelial-to-mesenchymal transition; Gli1, Glioma associated oncogene 1; Gli2, Glioma associated oncogene 2; GPx, Glutathione peroxidase; GSH, Reduced glutathione; HSCs, Hepatic stellate cells; HMG CoA, 3-hydroxy-3-methylglutaryl coenzyme A; HSP70, Heat shock protein 70; HSP90, Heat shock protein 90; MDA, Malondialdehyde; PDGF, Platelet derived growth factor; ROS, Reactive oxygen species; Shh, Sonic hedgehog; SMO, Smoothened; SMVT, Simvastatin; SOD, Superoxide dismutase; TAA, Thioacetamide; TC, Total cholesterol; TGF-β, Transforming growth factor beta; TG, Triglycerides; TIMP-1, Tissue inhibitor of metalloproteinase 1; TNF-α, Tumor necrosis factor alpha; γ-GT, Gamma glutamyl transferase.

## References

[B1] Abd El-Fattah E. E. Saber S. Youssef M. E. Eissa H. El-Ahwany E. Amin N. A. (2022). AKT-ampkα-mTOR-dependent HIF-1α activation is a new therapeutic target for cancer treatment: a novel approach to repositioning the antidiabetic drug sitagliptin for the management of hepatocellular carcinoma. Front. Pharmacol. 12. 10.3389/fphar.2021.720173 PMC8790251 35095479

[B2] Asrani S. K. Devarbhavi H. Eaton J. Kamath P. S. (2019). Burden of liver diseases in the world. J. Hepatol. 70, 151–171. 10.1016/j.jhep.2018.09.014 30266282

[B3] Baytan S. H. Alkanat M. Okuyan M. Ekinci M. Gedikli E. Ozeren M. (2008). Simvastatin impairs spatial memory in rats at a specific dose level. Tohoku J. Exp. Med. 214, 341–349. 10.1620/tjem.214.341 18441510

[B4] Beaven S. W. Tontonoz P. (2006). Nuclear receptors in lipid metabolism: targeting the heart of dyslipidemia, Annu. Rev. Med. 57 **,** 313–329. 10.1146/annurev.med.57.121304.131428 16409152

[B5] Berumen J. Baglieri J. Kisseleva T. Mekeel K. (2021). Liver fibrosis: pathophysiology and clinical implications, Liver Fibros. Pathophysiol. Clin. Implic. 13 **,** e1499. 10.1002/wsbm.1499 PMC9479486 32713091

[B6] Briscoe J. Thérond P. P. (2013). The mechanisms of Hedgehog signalling and its roles in development and disease. Nat. Rev. Mol. Cell Biol. 14, 416–429. 10.1038/nrm3598 23719536

[B7] Brown A. J. (2007). Cholesterol, statins and cancer. Clin. Exp. Pharmacol. Physiology 34, 135–141. 10.1111/j.1440-1681.2007.04565.x 17250629

[B8] Chatrath H. Vuppalanchi R. Chalasani N. (2012). Dyslipidemia in patients with nonalcoholic fatty liver disease. Semin. Liver Dis. 32, 22–29. 10.1055/s-0032-1306423 22418885 PMC3654545

[B9] Chen Y.-Y. Chen X.-G. Zhang S. (2022). Druggability of lipid metabolism modulation against renal fibrosis. Acta Pharmacol. Sin. 43, 505–519. 10.1038/s41401-021-00660-1 33990764 PMC8888625

[B10] Dutta R. K. Jun J. Du K. Diehl A. M. (2023). Hedgehog signaling: implications in liver pathophysiology. Semin. Liver Dis. 43, 418–428. 10.1055/a-2187-3382 37802119

[B11] Elmorsy E. A. Saber S. Hamad R. S. Abdel-Reheim M. A. Nadwa E. H. Alibrahim A. O. E. (2024). Modulating the HSP90 control over NFκB/NLRP3/Caspase-1 axis is a new therapeutic target in the management of liver fibrosis: insights into the role of TAS-116 (Pimitespib). Life Sci. 354, 122966. 10.1016/j.lfs.2024.122966 39147320

[B12] Friedman S. L. (2003). Liver fibrosis -- from bench to bedside. J. Hepatol. 38 (Suppl. 1), S38–S53. 10.1016/s0168-8278(02)00429-4 12591185

[B13] Gordon R. E. Zhang L. Peri S. Kuo Y. M. Du F. Egleston B. L. (2018). Statins synergize with hedgehog pathway inhibitors for treatment of medulloblastoma. Clin. Cancer Res. 24, 1375–1388. 10.1158/1078-0432.CCR-17-2923 29437795 PMC5856627

[B14] Hajovsky H. Hu G. Koen Y. Sarma D. Cui W. Moore D. S. (2012). Metabolism and toxicity of thioacetamide and thioacetamide S-oxide in rat hepatocytes. Chem. Res. Toxicol. 25, 1955–1963. 10.1021/tx3002719 22867114 PMC3444651

[B15] Henderson J. M. Polak N. Chen J. Roediger B. Weninger W. Kench J. G. (2018). Multiple liver insults synergize to accelerate experimental hepatocellular carcinoma. Sci. Rep. 8, 10283. 10.1038/s41598-018-28486-8 29980757 PMC6035229

[B16] Horn P. Tacke F. (2024). Metabolic reprogramming in liver fibrosis. Cell Metab. 36, 1439–1455. 10.1016/j.cmet.2024.05.003 38823393

[B17] Hu A. Song B.-L. (2019). The interplay of Patched, Smoothened and cholesterol in Hedgehog signaling. Curr. Opin. Cell Biol. 61, 31–38. 10.1016/j.ceb.2019.06.008 31369952

[B18] Hu L. Lin X. Lu H. Chen B. Bai Y. (2015). An overview of hedgehog signaling in fibrosis. Mol. Pharmacol. 87, 174–182. 10.1124/mol.114.095141 25395043

[B19] Ianevski A. Giri A. K. Aittokallio T. (2022). SynergyFinder 3.0: an interactive analysis and consensus interpretation of multi-drug synergies across multiple samples. Nucleic Acids Res. 50, W739–W743. 10.1093/nar/gkac382 35580060 PMC9252834

[B20] Ishak K. Baptista A. Bianchi L. Callea F. De Groote J. Gudat F. (1995). Histological grading and staging of chronic hepatitis. J. Hepatology 22, 696–699. 10.1016/0168-8278(95)80226-6 7560864

[B21] Kalonia H. Kumar P. Kumar A. (2011). Comparative neuroprotective profile of statins in quinolinic acid induced neurotoxicity in rats. Behav. Brain Res. 216, 220–228. 10.1016/j.bbr.2010.07.040 20696189

[B22] Keshk W. A. Ibrahim M. A. Shalaby S. M. Zalat Z. A. Elseady W. S. (2020). Redox status, inflammation, necroptosis and inflammasome as indispensable contributors to high fat diet (HFD)-induced neurodegeneration; Effect of N-acetylcysteine (NAC). Archives Biochem. Biophysics 680, 108227. 10.1016/j.abb.2019.108227 31838118

[B23] Kisseleva T. Brenner D. (2021). Molecular and cellular mechanisms of liver fibrosis and its regression. Nat. Rev. Gastroenterology and Hepatology 18, 151–166. 10.1038/s41575-020-00372-7 33128017

[B24] Kramann R. (2016). Hedgehog Gli signalling in kidney fibrosis. Nephrol. Dial. Transpl. 31, 1989–1995. 10.1093/ndt/gfw102 27229466

[B25] Krenkel O. Tacke F. (2017). Liver macrophages in tissue homeostasis and disease. Nat. Rev. Immunol. 17, 306–321. 10.1038/nri.2017.11 28317925

[B26] Lauth M. Rohnalter V. Bergström A. Kooshesh M. Svenningsson P. Toftgård R. (2010). Antipsychotic drugs regulate hedgehog signaling by modulation of 7-dehydrocholesterol reductase levels. Mol. Pharmacol. 78, 486–496. 10.1124/mol.110.066431 20558592

[B27] Li L. Wang L. You Q.-D. Xu X.-L. (2020). Heat shock protein 90 inhibitors: an update on achievements, challenges, and future directions. J. Med. Chem. 63, 1798–1822. 10.1021/acs.jmedchem.9b00940 31663736

[B28] Liu X.-N. Wang S. Yang Q. Wang Y.-J. Chen D.-X. Zhu X.-X. (2015). ESC reverses epithelial mesenchymal transition induced by transforming growth factor-β via inhibition of Smad signal pathway in HepG2 liver cancer cells. Cancer Cell Int. 15, 114. 10.1186/s12935-015-0265-2 26692820 PMC4676109

[B29] Lomba A. Milagro F. I. García-Díaz D. F. Marti A. Campión J. Martínez J. A. (2010). Obesity induced by a pair-fed high fat sucrose diet: methylation and expression pattern of genes related to energy homeostasis. Lipids Health Dis. 9, 60. 10.1186/1476-511X-9-60 20534152 PMC2909242

[B30] Lotersztajn S. Julien B. Teixeira-Clerc F. Grenard P. Mallat A. (2005). Hepatic fibrosis: molecular mechanisms and drug targets. Annu. Rev. Pharmacol. Toxicol. 45, 605–628. 10.1146/annurev.pharmtox.45.120403.095906 15471534

[B31] Malhotra P. Gill R. K. Saksena S. Alrefai W. A. (2020). Disturbances in cholesterol homeostasis and non-alcoholic fatty liver diseases, Front. Med. 7, 467, 10.3389/fmed.2020.00467 PMC7492531 32984364

[B32] Mehendale H. M. Chilakapati J. (2010). “9.29 - thioacetamide,” in Comprehensive toxicology. Editor Mcqueen C. A. (Oxford: Elsevier), 627–638.

[B33] Mihara M. Uchiyama M. (1978). Determination of malonaldehyde precursor in tissues by thiobarbituric acid test. Anal. Biochem. 86, 271–278. 10.1016/0003-2697(78)90342-1 655387

[B34] Mohamadin A. M. Elberry A. A. Abdel Gawad H. S. Morsy G. M. Al-Abbasi F. A. (2011). Protective effects of simvastatin, a lipid lowering agent, against oxidative damage in experimental diabetic rats. J. Lipids 2011, 167958. 10.1155/2011/167958 22191036 PMC3236494

[B35] Mohammed O. A. Abdel-Reheim M. A. Alamri M. M. S. Alfaifi J. Adam M. I. E. Saleh L. A. (2023a). STA9090 as a potential therapeutic agent for liver fibrosis by modulating the HSP90/tβrii/proteasome interplay: novel insights from *in vitro* and *in vivo* investigations. Pharmaceuticals 16, 1080. 10.3390/ph16081080 37630994 PMC10459039

[B36] Mohammed O. A. Abdel-Reheim M. A. Alamri M. M. S. Alfaifi J. Adam M. I. E. Saleh L. A. (2023b). STA9090 as a potential therapeutic agent for liver fibrosis by modulating the HSP90/tβrii/proteasome interplay: novel insights from *in vitro* and *in vivo* investigations. Pharmaceuticals 16, 1080. [Online]. 10.3390/ph16081080 37630994 PMC10459039

[B37] Mourad A. a.E. Khodir A. E. Saber S. Mourad M. a.E. (2021). Novel potent and selective DPP-4 inhibitors: design, synthesis and molecular docking study of dihydropyrimidine phthalimide hybrids. Pharmaceuticals 14, 144. 10.3390/ph14020144 33670273 PMC7918823

[B38] Müsch A. (2014). The unique polarity phenotype of hepatocytes. Exp. Cell Res. 328, 276–283. 10.1016/j.yexcr.2014.06.006 24956563 PMC4254207

[B39] Nasr M. Kira A. Y. Saber S. Essa E. A. El-Gizawy S. A. (2023). Lactosylated chitosan nanoparticles potentiate the anticancer effects of telmisartan *in vitro* and in a N-Nitrosodiethylamine-Induced mice model of hepatocellular carcinoma. Mol. Pharm. 20, 4758–4769. 10.1021/acs.molpharmaceut.3c00542 37585079

[B40] Omenetti A. Choi S. Michelotti G. Diehl A. M. (2011). Hedgehog signaling in the liver. J. Hepatol. 54, 366–373. 10.1016/j.jhep.2010.10.003 21093090 PMC3053023

[B41] Pearl L. H. Prodromou C. (2006). Structure and mechanism of the Hsp90 molecular chaperone machinery. Annu. Rev. Biochem. 75, 271–294. 10.1146/annurev.biochem.75.103004.142738 16756493

[B42] Reddy G. K. Enwemeka C. S. (1996). A simplified method for the analysis of hydroxyproline in biological tissues. Clin. Biochem. 29, 225–229. 10.1016/0009-9120(96)00003-6 8740508

[B43] Saber S. El-Fattah E. E. A. Abdelhamid A. M. Mourad A. a.E. Hamouda M. a.M. Elrabat A. (2023a). Innovative challenge for the inhibition of hepatocellular carcinoma progression by combined targeting of HSP90 and STAT3/HIF-1α signaling. Biomed. and Pharmacother. 158, 114196. 10.1016/j.biopha.2022.114196 36916405

[B44] Saber S. Hasan A. M. Mohammed O. A. Saleh L. A. Hashish A. A. Alamri M. M. S. (2023b). Ganetespib (STA-9090) augments sorafenib efficacy via necroptosis induction in hepatocellular carcinoma: implications from preclinical data for a novel therapeutic approach. Biomed. and Pharmacother. 164, 114918. 10.1016/j.biopha.2023.114918 37216705

[B45] Sasaki H. Hui C. Nakafuku M. Kondoh H. (1997). A binding site for Gli proteins is essential for HNF-3beta floor plate enhancer activity in transgenics and can respond to Shh *in vitro* . Development 124, 1313–1322. 10.1242/dev.124.7.1313 9118802

[B46] Scutigliani E. M. Liang Y. Ijff M. Rodermond H. Mei X. Korver M. P. (2022). Evaluation of the heat shock protein 90 inhibitor ganetespib as a sensitizer to hyperthermia-based cancer treatments. Cancers (Basel). 14, 5250. 10.3390/cancers14215250 36358669 PMC9654690

[B47] Seki E. De Minicis S. Osterreicher C. H. Kluwe J. Osawa Y. Brenner D. A. (2007). TLR4 enhances TGF-beta signaling and hepatic fibrosis. Nat. Med. 13, 1324–1332. 10.1038/nm1663 17952090

[B48] Shalaby R. H. Rashed L. A. Foda F. M. Kamel A. H. Adel R. M. (2017). Effect of simvastatin on male albino rats, cytogenetic and histochemical studies. J. Sci. Res. Sci. 34, 68–84. 10.21608/jsrs.2018.12756

[B49] Sironi L. Cimino M. Guerrini U. Calvio A. M. Lodetti B. Asdente M. (2003). Treatment with statins after induction of focal ischemia in rats reduces the extent of brain damage. Arteriosclerosis, Thrombosis, Vasc. Biol. 23, 322–327. 10.1161/01.atv.0000044458.23905.3b 12588778

[B50] Stål P. (2015). Liver fibrosis in non-alcoholic fatty liver disease - diagnostic challenge with prognostic significance. World J. Gastroenterol. 21, 11077–11087. 10.3748/wjg.v21.i39.11077 26494963 PMC4607906

[B51] Stirnimann J. Stirnimann G. (2019). Nutritional challenges in patients with advanced liver cirrhosis. J. Clin. Med. 8, 1926. 10.3390/jcm8111926 31717529 PMC6912781

[B52] Stopa M. Anhuf D. Terstegen L. Gatsios P. Gressner A. M. Dooley S. (2000). Participation of Smad2, Smad3, and Smad4 in Transforming Growth Factor β (TGF-β)-induced Activation of Smad7: the tf-β response element of the promoter requires functional smad binding element and e-box sequences for transcriptional regulation *. J. Biol. Chem. 275, 29308–29317. 10.1074/jbc.M003282200 10887185

[B53] Summer R. Mora A. L. (2019). Lipid metabolism: a new player in the conundrum of lung fibrosis. Am. J. Respir. Cell Mol. Biol. 61, 669–670. 10.1165/rcmb.2019-0098ED 31499006 PMC6890398

[B54] Syn W. K. Jung Y. Omenetti A. Abdelmalek M. Guy C. D. Yang L. (2009). Hedgehog-mediated epithelial-to-mesenchymal transition and fibrogenic repair in nonalcoholic fatty liver disease. Gastroenterology 137, 1478–1488. 10.1053/j.gastro.2009.06.051 19577569 PMC2757536

[B55] Taipale M. Jarosz D. F. Lindquist S. (2010). HSP90 at the hub of protein homeostasis: emerging mechanistic insights. Nat. Rev. Mol. Cell Biol. 11, 515–528. 10.1038/nrm2918 20531426

[B56] Talreja O. Kerndt C. C. Cassagnol M. (2024). “Simvastatin,” in *StatPearls*. Treasure Island (FL) ineligible companies. Disclosure: connor Kerndt declares no relevant financial relationships with ineligible companies. Disclosure: manouchkathe Cassagnol declares no relevant financial relationships with ineligible companies (Treasure Island, FL: StatPearls Publishing LLC).

[B57] Tan Z. Sun H. Xue T. Gan C. Liu H. Xie Y. (2021). Liver fibrosis: therapeutic targets and advances in drug therapy, 9. 10.3389/fcell.2021.730176 PMC8490799 34621747

[B58] Taniguchi H. Hasegawa H. Sasaki D. Ando K. Sawayama Y. Imanishi D. (2014). Heat shock protein 90 inhibitor NVP-AUY922 exerts potent activity against adult T-cell leukemia-lymphoma cells. Cancer Sci. 105, 1601–1608. 10.1111/cas.12540 25263741 PMC4317953

[B59] Tsai Y. C. Lam K. K. Peng Y. J. Lee Y. M. Yang C. Y. Tsai Y. J. (2016). Heat shock protein 70 and AMP-activated protein kinase contribute to 17-DMAG-dependent protection against heat stroke. J. Cell Mol. Med. 20, 1889–1897. 10.1111/jcmm.12881 27241357 PMC5020632

[B60] Tsuchida T. Friedman S. L. (2017). Mechanisms of hepatic stellate cell activation. Nat. Rev. Gastroenterology and Hepatology 14, 397–411. 10.1038/nrgastro.2017.38 28487545

[B61] Tuladhar R. Yarravarapu N. Ma Y. Zhang C. Herbert J. Kim J. (2019). Stereoselective fatty acylation is essential for the release of lipidated WNT proteins from the acyltransferase Porcupine (PORCN). J. Biol. Chem. 294, 6273–6282. 10.1074/jbc.RA118.007268 30737280 PMC6484125

[B62] Wiering L. Subramanian P. Hammerich L. (2023). Hepatic stellate cells: dictating outcome in nonalcoholic fatty liver disease. Cell Mol. Gastroenterol. Hepatol. 15, 1277–1292. 10.1016/j.jcmgh.2023.02.010 36828280 PMC10148161

[B63] Wu S. Wang X. Xing W. Li F. Liang M. Li K. (2023). An update on animal models of liver fibrosis. Front. Med. (Lausanne) 10, 1160053. 10.3389/fmed.2023.1160053 37035335 PMC10076546

[B64] Yanguas S. C. Cogliati B. Willebrords J. Maes M. Colle I. Van Den Bossche B. (2016). Experimental models of liver fibrosis. Arch. Toxicol. 90, 1025–1048. 10.1007/s00204-015-1543-4 26047667 PMC4705434

[B65] Yauch R. L. Dijkgraaf G. J. P. Alicke B. Januario T. Ahn C. P. Holcomb T. (2009). Smoothened mutation confers resistance to a Hedgehog pathway inhibitor in medulloblastoma. Smoothened Mutat. Confers Resist. a Hedgehog Pathw. Inhibitor Medulloblastoma 326, 572–574. 10.1126/science.1179386 PMC5310713 19726788

[B66] Yeung D. (2015). Does a high-fat diet cause inflammation in female rat brain?. Waterloo, Canada: University of Waterloo.

[B67] Yildiz M. K. Okan I. Dursun N. Bas G. Alimoglu O. Kaya B. (2014). Effect of orally administered simvastatin on prevention of postoperative adhesion in rats. Int. J. Clin. Exp. Med. 7, 405–10. 24600496 PMC3931595

[B68] Youssef M. E. El-Azab M. F. Abdel-Dayem M. A. Yahya G. Alanazi I. S. Saber S. (2022). Electrocardiographic and histopathological characterizations of diabetic cardiomyopathy in rats. Environ. Sci. Pollut. Res. 29, 25723–25732. 10.1007/s11356-021-17831-6 34845640

[B69] Zhao M. Wang L. Wang M. Zhou S. Lu Y. Cui H. (2022). Targeting fibrosis: mechanisms and clinical trials. Signal Transduct. Target. Ther. 7, 206. 10.1038/s41392-022-01070-3 35773269 PMC9247101

